# GeSnOI mid-infrared laser technology

**DOI:** 10.1038/s41377-021-00675-7

**Published:** 2021-11-17

**Authors:** Binbin Wang, Emilie Sakat, Etienne Herth, Maksym Gromovyi, Andjelika Bjelajac, Julien Chaste, Gilles Patriarche, Philippe Boucaud, Frédéric Boeuf, Nicolas Pauc, Vincent Calvo, Jérémie Chrétien, Marvin Frauenrath, Alexei Chelnokov, Vincent Reboud, Jean-Michel Hartmann, Moustafa El Kurdi

**Affiliations:** 1 Université Paris-Saclay, CNRS, C2N, 10 boulevard Thomas Gobert, 91120 Palaiseau, France; 2 Université Côte d’Azur, CNRS, CRHEA, Rue Bernard Grégory, 06905 Sophia-Antipolis, France; 3 STMicroelectronics, Rue Jean Monnet, 38054 Crolles, France; 4 Université Grenoble Alpes, CEA, IRIG-DePhy, 17 rue des Martyrs, 38000 Grenoble, France; 5 Université Grenoble Alpes, CEA, Leti, 17 rue des Martyrs, 38000 Grenoble, France

**Keywords:** Semiconductor lasers, Microresonators, Silicon photonics, Integrated optics

## Abstract

GeSn alloys are promising materials for CMOS-compatible mid-infrared lasers manufacturing. Indeed, Sn alloying and tensile strain can transform them into direct bandgap semiconductors. This growing laser technology however suffers from a number of limitations, such as poor optical confinement, lack of strain, thermal, and defects management, all of which are poorly discussed in the literature. Herein, a specific GeSn-on-insulator (GeSnOI) stack using stressor layers as dielectric optical claddings is demonstrated to be suitable for a monolithically integration of planar Group-IV semiconductor lasers on a versatile photonic platform for the near- and mid-infrared spectral range. Microdisk-shape resonators on mesa structures were fabricated from GeSnOI, after bonding a Ge_0.9_Sn_0.1_ alloy layer grown on a Ge strain-relaxed-buffer, itself on a Si(001) substrate. The GeSnOI microdisk mesas exhibited significantly improved optical gain as compared to that of conventional suspended microdisk resonators formed from the as-grown layer. We further show enhanced vertical out-coupling of the disk whispering gallery mode in-plane radiation, with up to 30% vertical out-coupling efficiency. As a result, the GeSnOI approach can be a valuable asset in the development of silicon-based mid-infrared photonics that combine integrated sources in a photonic platform with complex lightwave engineering.

## Introduction

Low-cost and CMOS-compatible Si-based photonic technologies, like Silicon-On-Insulator (SOI), has enabled significant advances for on-chip optical processing in the near-infrared (IR) wavelength range, especially for high-speed detection and modulation of optical signals^1^. One of its major drawbacks, though, is the lack of monolithically integrated group-IV lasers. Indeed, group-IV alloys are indirect bandgap semiconductors. To compensate for the lack of such laser sources, strong efforts were devoted these past few years to the integration of III–V compounds with high lasing performances to boost silicon photonic technologies^2^. It was particularly true for telecom applications in the near-infrared wavelength range. So far, III–V lasers are the most standard and reliable light sources on silicon despite their high manufacturing cost and their complex integration in Si CMOS-compatible manufacturing processes^3,4^. GeSn semiconductor alloys, with a direct bandgap for tin contents larger than 7% (for strain-free materials)^5^ and which are compatible with large-scale and low-cost silicon processing and manufacturing tools, are promising for low-cost lasers^6–8^. In addition, GeSn alloys have a narrow bandgap as compared to Ge and are thus suitable to shift the photonic wavelength from near-IR to mid-IR where many application domains exist: biochemical detection, gas monitoring, and thermal imaging. Integrating GeSn on silicon opens up new application fields for Si photonics^9,10^.

In practice, the epitaxial growth of GeSn on silicon is quite challenging. The very low thermal equilibrium solubility of Sn in Ge, of only 1%, requires the development of metastable growth methods to increase the Sn content above 7%. Moreover, the very high lattice mismatch between GeSn and silicon makes their growth tricky. The use of Ge strain-relaxed buffer (Ge SRB) on silicon is the only approach used so far yielding high enough material quality for lasing. However, it still faces major issues^11–13^. For example, the active GeSn layers are usually grown beyond their critical thickness for plastic relaxation of the compressive strain at the GeSn/Ge interface^14^. Compressive strains are unfavorable since they worsen the optical gain properties of GeSn alloys, and, specifically, their sustainability with increasing temperature. Indeed, they reduce the energy barrier between the direct *E*_Γ_ and indirect *E*_L_ conduction band valleys (the so-called material directness parameter)^15^. Compressive strain in pseudomorphically grown GeSn alloys can turn, even for high Sn contents, the bandgap from direct to indirect, preventing gain and lasing^16^. Moreover, the relaxation of this compressive strain during the growth of really thick layers results in the formation of a dense array of misfit dislocations network close to the interface, at the bottom of the optically active region^5,8^. Thus, many strategies have been employed these past few years to address this compressive strain issue while attempting to reach the highest material quality possible: growth of thick GeSn layers with gradually increasing tin content in order to limit the propagation of misfit defects into the bulk of the GeSn layer, use of SiGeSn barriers to confine the carriers away from the interfacial defects or, even, multi-quantum wells^11,17–21^. All of those methods are based on in situ strain and defects management and have thus limited flexibility due to metastable growth mechanism constraints.

In this framework, the active injection of tensile strain into GeSn, via external stressor layers, is theoretically and experimentally known to offer further degrees of freedom to tune the strain and consequently the electronic band structure^22^. It also opens up new possibilities of enhancing gain properties and tuning the laser wavelength^23,24^. Mainstream technological approaches for strain management call upon external stressor layers such as SiN^25–27^ or mechanical pulling of microbridges by external pads^28–30^. They were mainly used for pure germanium, up to now. In both cases, layers should be suspended in order to increase the tensile stress injection and reach high enough optical confinement. Indeed, blanket GeSn on Ge SRB structures suffers from a low optical index contrast between GeSn and Ge, resulting in low optical confinement factors in the active region^31^. The active layer thickness has then to be increased in order to reach values in the range of the operating wavelength, e.g. 1 µm^6^, making external tensile strain injection more challenging. Suspended microdisk (MD) cavities or microbridges were thus the main structures evaluated up to now for such strained laser devices^22,32^.

In this work, we show that GeSnOI stacks obtained through the bonding of GeSn active layers tackle all the above-mentioned issues: lattice mismatch interface defects management, compressive/tensile strain management, and optical confinement. This is demonstrated through a systematic comparison of two structures with equivalent optical confinements: a suspended MD cavity fabricated from a GeSn layer on a Ge SRB, as in the existing literature, and another one, fabricated using the GeSnOI approach, with the use of specific SiN stressor films as insulating layers and with a simple disk-shape mesa as the cavity. The SiN layers used for tensile strain engineering yielded high index contrast with GeSn, which was required for optical confinement. After the bonding of the GeSn layer, the dense array of misfit dislocations close to the GeSn/Ge interface was removed thanks to a simple etching step, resulting in a better active layer quality that should result in an improved carrier injection efficiency. Another advantage offered by such bonded structures was the possibility of having the GeSn layer standing on an aluminum layer, resulting in a better heat dissipation^27,33^. Moreover, such disk cavities being bonded to the substrate without any under-etching, it was also possible to downscale their diameters to 3 µm, which was not feasible with as-grown GeSn-MD (lowest achievable diameters of 5 µm) for mechanical and thermal robustness reasons (the Ge pedestals cannot be too thin). Last but not least, the MD-shape mesa cavity obtained with this new approach also offered the possibility of managing the whispering gallery modes (WGM) in-plane radiation that made the collection of the laser light tricky. A specific design with circular diffraction grating around the MD was proposed to redirect the emission pattern vertically without changing the MD design or the lasing characteristics.

## Results

**Fig. 1:**
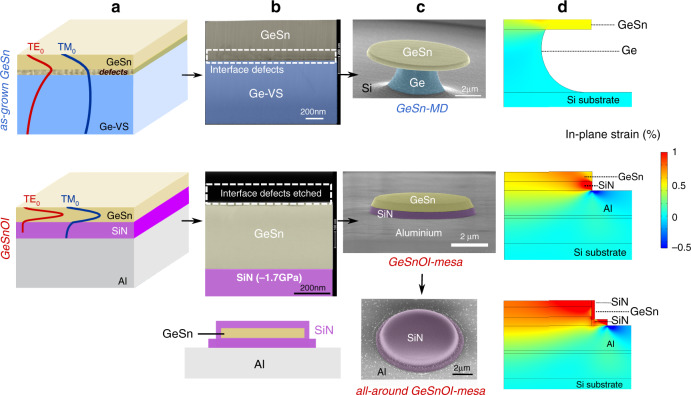
Design and fabrication of GeSnOI planar cavities. **a** Schematic diagram of as-grown GeSn on Ge SRB and GeSnOI stacks together with TE and TM mode profiles of a confined optical wave at a 2.4 µm wavelength. More details are given in the SI (Fig. S3). **b** X-TEM images of as-grown GeSn on Ge SRB and of GeSnOI stacks. **c** Scanning electron microscopy (SEM) images of GeSn and GeSnOI and all-around strained microdisk cavities. A cross-sectional schematic representation of the GeSnOI mesa fully cladded with SiN can be found on the left of the bottom SEM image (**d**) in-plane strain variation, analyzed by Finite Element modeling (FEM), of GeSn and GeSnOI microdisks with 7 µm diameters that due to the different designs and processing adopted. Here we plot the strain relative to initial residual post-growth compressive strain of −0.5% for the GeSn layer. The SiN layer was computed with initial compressive stress that can relax, thus inducing positive strain variation^26^

We start with a presentation of the two kinds of structures. Both were based on a 500-nm-thick GeSn layer with 10.5% of Sn grown on a 2.5-μm-thick Ge SRB, itself on a 200 mm Si (001) wafer (Fig. 1a). The growth was performed in an industrial reduced-pressure chemical vapor deposition (RP-CVD). The as-grown GeSn layer had a residual compressive strain of −0.5% as estimated from X-ray diffraction reciprocal space mapping^5^. For such GeSn layer on Ge SRB, the critical thickness is around 100 nm. A dense array of misfit dislocations loops occurs up to a depth of typically 100 nm from the GeSn/Ge interface, can clearly be seen by cross-sectional transmission electron microscopy (X-TEM) (Fig. 1b), a zoomed view is provided in the SI (Fig. S1).

The optical index contrast is low in such a GeSn on the Ge SRB stack, resulting in a poor mode overlap with the active GeSn layer (Fig. 1a). The transverse electric (TE)-polarized propagating wave indeed has an overlap of only 37% with the GeSn active layer and its electrical field maximum is close indeed to the dislocated interfacial region. Similarly, a blanket GeSn on Ge stack provides a very weak transverse magnetic (TM)-polarized light confinement, with an overlap of only 11% with the GeSn active layer. To circumvent such bad optical confinements, an isotropic under-etching of the Ge SRB is generally performed in order to obtain suspended GeSn-MD laser devices (Fig. 1c). In this configuration, the WGM situated at the edges of the disk experiment a GeSn/air optical index contrast which yields optical confinement factors of 95% and 78% for TE and TM, respectively.

**Fig. 2:**
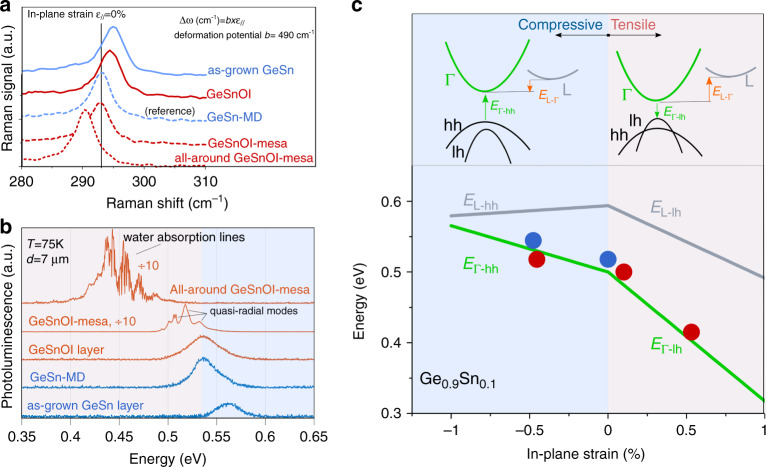
Optical analysis of strain. **a** Raman spectra of LO-phonon vibrations in the blanket, as-grown GeSn 10.5% on Ge SRB stacks, blanket GeSnOI stacks, GeSn-on-Ge SRB microdisks, GeSnOI mesas, and all-around GeSnOI mesas. The diameter of disks and mesa was 7 µm. **b** Photoluminescence spectra of GeSn layers as in (**a**) excited with cw optical pumping. **c** Direct and indirect bandgap energies of GeSn as a function of strain. Direct bandgap energies from PL measurements appear as blue circles for the as-grown GeSn 10.5% on Ge SRB stack and the GeSn-on-Ge SRB microdisks. Red circles show the PL energies for the blanket GeSnOI stack, the GeSnOI mesa, and the all-around GeSnOI mesa (from left to right). Full lines are calculated direct bandgap (green) and indirect bandgap (gray) energies as functions of strain using model as described in ref. ^5^. The top picture is a schematic scenario of band structure for compressive and tensile-strained GeSn

For the MD-shape mesa laser cavity, the approach was different: a piece of the very same GeSn on Ge SRB was bonded on a host Si substrate coated with aluminum and SiN layers to obtain the GeSnOI structure shown in Fig. 1b, c (process detailed in the section A of the SI). The aluminum layer acted as a heat sink while the SiN layer acted as a stressor and provided optical confinement. After bonding, the Si substrate used to grow the Ge SRB was removed by selective etching. The Ge SRB and defects at the GeSn/Ge interface were then removed by another etching step. The final GeSn layer was then thinned from the as-grown 500 nm down to 400 nm, to get rid of the defective interfacial layer, which was then on top, and benefit from improved crystalline quality. The GeSn/SiN index contrast was high enough to provide excellent optical confinement for TE and TM-polarized modes: optical confinement factors were 93% for TE and 77% for TM, e.g., values very close to that in GeSn MDs (Fig. 1c). This enabled us to suppress the impact of confinement on the optical gain when comparing the lasing performances of conventional GeSn/Ge MDs and MD-shape mesas based on bonded GeSnOI. Confining the modes in a few hundreds of nm-thick-GeSn layer was suitable for homogeneously distributed external tensile strain injection (Fig. 1d). The SiN insulator was initially compressively stressed, by typically −1.7 GPa. The strain was partly released and transferred to the GeSn layer after patterning the GeSnOI layers into MD-shape mesas, as shown by the FEM analysis. The GeSn layer had an initial compressive strain of −0.5% which vanished after the bottom SiN layer had partially released its built-in stress. This led to a very moderately tensile-strained structure (Fig. 1d). To further increase the tensile strain, an additional top SiN stressor layer can be deposited, resulting in an all-around structure. The tensile strain is then homogeneously distributed in the GeSn disk, as shown in Fig. 1d. An SiN stressor layer could also be deposited on top of the GeSn-on-Ge SRB MD, as proposed recently^24,34^. The distributed tensile strain would be very inhomogeneous, then, which would not be favorable for lasing, as discussed in the SI (section F). Raman analysis of the strain (Fig. 2a) was performed using the strain and alloy potentials of ref. ^5^. To identify the influence of strain on the band structure, we have performed a continuous wave (cw) photoluminescence (PL) analysis at 75 K under 1550 nm wavelength optical pumping. Different configurations, blanket GeSn on Ge SRB stacks, GeSn-on-Ge SRB MDs, blanket GeSnOI stacks, GeSnOI mesas and all-around GeSnOI mesas, were studied under the same experimental conditions, as shown in Fig. 2.

The suspended GeSn MDk has a PL emission red-shifted by 25 meV with respect to the PL emission from the blanket, unprocessed GeSn layer, which is due to compressive strain relaxation through the under-etching of the Ge SRB at the disk edges. The continuous wave PL spectrum of the GeSnOI mesa has red-shifted by 18 meV only with respect to the PL spectrum of the strain-relaxed GeSn-MD (without SiN stressor). This indicates that the mesa is very weakly tensile-strained, as confirmed by Raman analysis which gives only around 0.1% of in-plane strain, in line with FEM analysis. On the other hand, the all-around GeSnOI disk-shape mesa has a 100 meV red-shifted emission compared to the GeSn-MD. An injected equivalent biaxial tensile strain of 0.55% was measured with Raman spectroscopy, once again in good agreement with FEM analysis. We have plotted in Fig. 2c the bandgap energies extracted from PL spectra as a function of the strain measured by Raman spectroscopy. Full curves are theoretical direct bandgap Γ-hh (Γ-lh) and indirect bandgap L-hh (L-lh) energies as functions of compressive (or tensile) strain^5^. A quite good agreement is obtained with experimental results (points in blue or red). The SiN stressor thus enabled us to modify the strain state of our GeSn 10.5% alloy, from a −0.5% compressive state to a 0.55% tensile state, e.g., a shift by ~ +1% of the strain. Band structure engineering was thus feasible without changing the Sn content.

To conclude this section, let us summarize the improvements provided by MD-shape mesas etched in bonded GeSnOI stacks as compared to suspended GeSn MDs. First, a GeSnOI disk-shape mesa has no interfacial defects anymore, while the suspended GeSn-MD still has defects at the pedestal’s GeSn/Ge interface. Second, the bonded structure is not under-etched and incorporates an aluminum layer which acts as a heat sink, enabling a better thermal management. The optical confinement factor of optical modes with the active GeSn layer is as good in GeSnOI mesas than in under-etched structures suspended in the air. Finally, a full cladding of the GeSn mesa with SiN insulator layers yields high levels of homogeneously distributed tensile strain, something which is not really feasible with a suspended GeSn-MD. In the latter case, the only practical option is to add a stressor on the top of the suspended GeSn layer^24,34^. Such configuration results in an inhomogeneous tensile strain which is not convenient for lasing (see the SI section). Let us now show, in the next sections, how these improvements enabled us to enhance lasing performances.

**Fig. 3:**
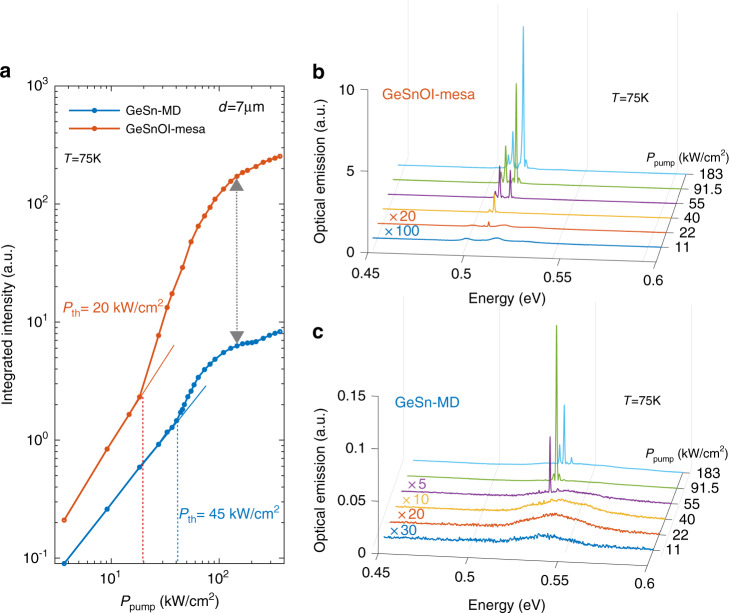
GeSnOI mesa versus as-grown GeSn microdisk laser. **a** Light–Light (L–L) curves of 7 µm diameter GeSn microdisks and GeSnOI mesas with 360-nm-thick GeSn at 75 K under pulsed pumping (**b**) and **c** PL spectra at 75 K for various pulsed excitation powers for the GeSnOI mesa and the GeSn-MD, respectively. Excitation levels are given by the peak power densities

Lasing performances were compared for the same 7 µm diameter mesas and MDs. The GeSnOI mesa has an active layer thickness of 360 nm, while the GeSn-MD active layer thickness is 500 nm. The analysis of the MD emission was performed with a 1550 nm wavelength pulsed optical pumping with a pulse width of 3.5 ns and a 25 MHz repetition rate. This illumination condition yielded high excitation densities while avoiding any significant thermal heating (SI section C). As shown in Fig. 3a–c, we observed clear lasing for mesas and MDs in the pulsed excitation regime. However, a two times lower threshold was obtained with the GeSnOI mesa (20 kW/cm^2^), as compared to the GeSn-MD (45 kW/cm^2^). In addition, the GeSnOI mesa had a peak intensity above lasing threshold which was 60 times higher than that of the GeSn-MD (Fig. 3b, c), while the spontaneous emission below threshold was typically four times more intense (Fig. 3a). We can note that the PL enhancement, below threshold with GeSnOI mesa due to the presence of the reflective Al layer with respect to the GeSn-MD, can be estimated to be 1.7, which is lower than the observed enhancement. Furthermore (i) the GeSnOI mesa absorbs less efficiently, by a factor of 1.7, the incident pump beam (as discussed below); (ii) the GeSnOI active layer is a factor of 1.4 thinner than the GeSn-MD and thus has less emitters in its volume. We can thus assume that the improved PL signal below the threshold, despite the given above consideration, goes to the sense that carrier losses from non-radiative recombination process are partly reduced with the GeSnOI mesa, which could also partly explain such gain dynamic and threshold improvements^35,36^. In previous work, we already demonstrated that the power density threshold in suspended GeSn MDs was one order of magnitude lower after the partial removal of interfacial defects from the MD edge region during the Ge under-etching^5^. However, interface defects above the Ge pillars were still present. Here, defects in GeSnOI mesas were removed from the whole surface, most likely explaining why power density thresholds were further reduced. The tensile strain in the GeSnOI mesa was small (only around 0.1%), with a limited impact on band structure and gain. We can note that the presence of the reflective Al layer, in the case of GeSnOI stack, may induce an enhancement of the pump beam absorption by reflection effect from the bottom Al layer. However depending on the SiN layer thickness, one may have constructive or destructive interference between the incident and the reflected pump beam, inside the active GeSn region. In our specific case, the GeSnOI stack dimensions were not favorable, and the GeSn active layer is positioned at a node of the electrical field distribution along the GeSn/SiN/Al stacking. We estimated that in this configuration, the absorbed power of incident pump is significantly larger here for the GeSn-MD case, by typically a factor 1.7. The absorption coefficient is estimated to be 30% for the GeSn-MD while the GeSnOI mesa have an absorption coefficient of 18%. We can thus assume that the improved lasing thresholds were not induced by enhanced pump power absorption in the GeSnOI mesa. However further improvement of the laser characteristics could be reached thanks to appropriate design of the GeSnOI stack, for example, by using an SiN layer thickness of 600 nm, instead of 400 nm as in the present case. With such SiN layer thickness the GeSn active layer would thus be positioned favorably at the antinode of the electrical field distribution of the pump such that one could reach a strongly improved efficiency of the pump absorption, typically by a factor of 4.5. In this case the absorption coefficient of the pump could reach 80%. It is additionally important to note here that the quality (*Q*) factor of the GeSnOI mesa is two times lower than that of the GeSn-MD, which should have increased the GeSnOI mesa’s lasing threshold. It is however lower than that of the GeSn-MD, illustrating the importance of defects removal to improve the gain. One can also observe in Fig. 2b that the GeSnOI mesa features multimodal lasing, which is the signature of spatial and spectral broadening of the gain, whereas the GeSn-MD has mostly a monomode lasing signature.

This can be explained by the presence of the Ge pedestal in the GeSn-MD. Indeed, the edges of the pedestal are 1.5–2 µm away from the MD periphery, resulting in optical losses for higher-order radial modes, i.e., those which have a higher overlap of the optical field with the pedestal. The *Q*-factor of these modes decreases and they are not able to lase. On the contrary, the same diameter GeSnOI mesa has the whole surface isolated from the substrate by the bottom SiN layer. Therefore, the higher-order radial modes do not incur any pedestal losses. This is also confirmed by the PL spectra of the GeSnOI mesa below threshold, with the presence of high-order radial mode resonances (denoted as quasi-radial modes in Fig. 2b), which were not observed for the GeSn-MD. The multimodal features thus could also be explained by a higher spatial gain broadening with the GeSnOI mesa.

**Fig. 4:**
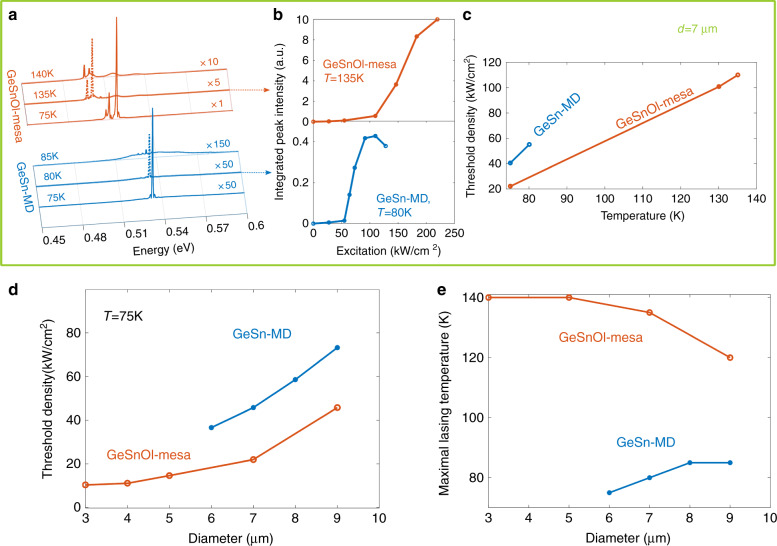
Improved lasing performances with GeSnOI. **a** Pulsed PL spectra of 7 µm diameter GeSn microdisks and GeSnOI mesas with 360-nm-thick GeSn above the threshold at 75 K (solid curves), at maximum lasing temperatures (dotted curves) and at temperatures 5 K higher (dashed curves). The laser intensities in GeSn MDs and GeSnOI mesas are the highest for 91.5 and 183 kW/cm^2^ excitation power densities, respectively. **b** L–L curves for GeSn MDs and GeSnOI mesas at their maximum lasing temperature, 80 and 135 K, respectively. **c** Temperature dependence of threshold densities for 7 µm diameter GeSn MDs and GeSnOI mesas. **d** Power density thresholds at 75 K for different diameter GeSn-MD and GeSnOI mesa lasers. **e** Highest lasing temperatures for different diameter GeSn MDs and GeSnOI mesas

The GeSnOI mesa gain also persists at higher temperature. Lasing spectra above threshold (Fig. 4a) along with L–L curves and lasing thresholds (Fig. 4b, c) at different temperatures clearly show the improvement when switching from 7 µm diameter GeSn MDs to GeSnOI mesas, with maximum lasing temperatures of 80 and 135 K, respectively. One can estimate, from threshold dependence with temperature of Fig. 4c, a *T*_0_ value of 40 K. This value can be associated with activation of non-radiative recombination process, and intervalley scattering that weaken the optical gain when increasing the temperature. The slope efficiency is as well better above threshold for GeSnOI mesas and this feature is attributed to higher quantum efficiency associated with better carrier distribution in the gain region.

Then, different diameter GeSn MDs and GeSnOI mesas were systematically studied to compare their lasing threshold and their maximum lasing temperature. Thresholds at 75 K are provided in Fig. 4d and maximum lasing temperatures in Fig. 4e, both as functions of the MDs diameter. We reproducibly obtained lower thresholds and higher maximum lasing temperature for GeSnOI mesas than for GeSn MDs.

A heat dissipation study was conducted for suspended GeSn MDs (see SI section C). It showed that thermal cooling strongly depended on the MD geometry. We did not observe lasing for MD diameters smaller than 6 µm with therefore very narrow Ge pedestals. Since the under-etching was the same for all diameters, MDs with the largest diameters had larger pedestals and evacuated heat more effectively. For GeSn-MD diameters between 7 and 9 µm, the temperature increase upon optical injection should be around 15 K for a 200 kW/cm^2^ excitation density in a pulsed regime, as used in the current experiments. Yet, MDs were not able to reach lasing at temperatures higher than 85 K. This maximum lasing temperature is similar to that in a previous work where MDs were fabricated from the very same GeSn 10.5% layer with equivalent disk diameters but smaller undercuts (1.5 µm instead of 2 µm here). Such reduced lasing temperatures are likely due to limited heat dissipation and gain decrease as the temperature increases.

**Fig. 5:**
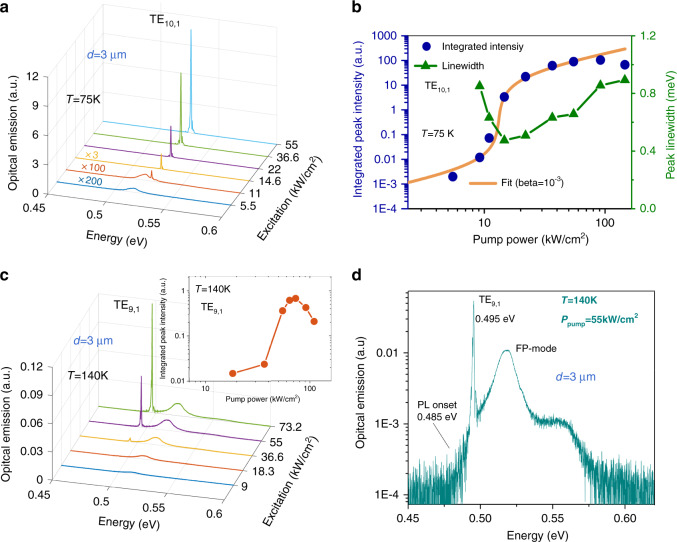
Down-scaled GeSnOI mesa laser. **a** Pulsed PL spectra of 3 µm diameter GeSnOI mesa with 360-nm-thick GeSn under various excitation densities at 75 K. **b** the L–L curve of the laser mode TE_10,1_ at 75 K in log–log scale and mode linewidth as a function of the pump power. **c** same as **a** at 140 K, the inset shows the L–L curve in a log–log scale for the lasing mode which is the TE_9,1_ in this case. **d** Emission spectrum above threshold at 140 K highlighting the TE_9,1_ lasing mode position with respect to the spontaneous emission. The spontaneous emission is itself strongly modulated by broad peak that stems from Fabry–Pérot (FP) resonance. These FP-modes emit more favorably out-of-plane and are thus better collected than the WGMs

In GeSnOI mesas, the maximum lasing temperature is 140 K, i.e., 55 K higher than in suspended GeSn MDs. Such an improvement is attributed to a higher crystalline quality, i.e., a lower number of non-radiative recombination channels making the temperature dependence of gain more robust, and to the increased heat dissipation. With such mesa structures, disk diameters can be shrunk down to 3 µm without degrading thermal dissipation upon optical pumping. A reduced size seems to help heat dissipation. Indeed, as seen in Fig. 4e, the lowest diameter mesa has the highest lasing temperatures. Such 3 µm diameter MD mesa also features the smallest lasing thresholds, only 12 kW/cm^2^ at 75 K and 40 kW/cm^2^ at 140 K (Fig. 5a, b).

**Fig. 6:**
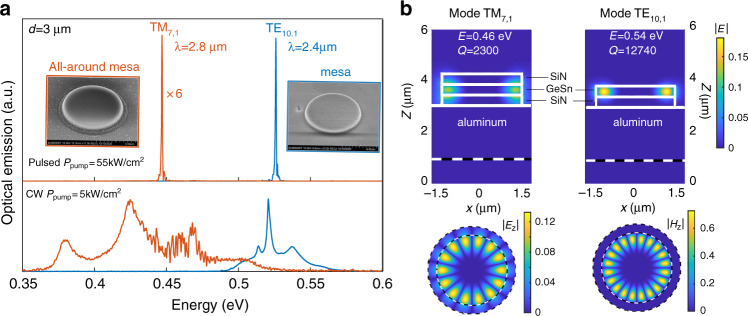
Tensile strain tuning of GeSnOI laser emission. **a** Spectra below and above threshold density for 3 µm diameter GeSnOI mesas with (red curves) or without (blue curves) top SiN stressors. In both cases, the active GeSn layer thickness was 400 nm. **b** Simulated optical fields and theoretical *Q*-factor values of TM_7,1_ and TE_10,1_ lasing modes for a tensile-strained GeSnOI mesa with a SiN stressor on top and a quasi-relaxed GeSnOI mesa without the top SiN stressor, respectively. Calculations have been performed with an aperiodic Fourier modal method dedicated to body-of-revolution objects^45^

Lasing thresholds decrease with the mesa or MD diameters. This was expected as smaller volume cavities have larger spontaneous coupling factors with the optical modes^36,37^. Here, the coupling factor *β* was found to be 10^−3^ from a fit of L–L curve of the lasing mode using laser rate equations (Fig. 5b). The 3 µm smallest diameter mesa indeed features single-mode lasing at 0.525 eV at 75 K, which is attributed to a TE_10,1_ WGM resonance. The sharp linewidth decrease at threshold for this mode allows for an estimate of an equivalent *Q*-factor value at transparency of typically 1000. A linewidth increase is observed above threshold due to thermal effects. This *Q*-factor value at transparency has to be compared with modeled values of *Q*-factor around 10,000–13,000 (see Fig. 6). Note that the measurements are performed in pulsed excitation and the linewidth might vary over the pulse duration, thus inducing larger experimental linewidth than modeled ones in the framework of steady-state regime. The differences may also stem from fabrication imperfections such as surface roughness of the GeSnOI layer after bonding (Fig. S1) as well as the sidewall roughness of the mesas. The modeled free spectral range is equal to 35 meV for the fundamental radial mode *n* = 1 in such small diameter mesas, which is of the same order of the spontaneous emission broadening. It is thus expected that such a small cavity has a reduced number of modes that overlap with gain, resulting in single-mode lasing as obtained on Fig. 5a–c. At 140 K, the laser emission peak has shifted by 30 meV to lower energy as compared to the laser emission peak at 75 K. Note that the bandgap energy, and thus the optical gain is expected to redshift by typically 10 meV when increasing temperature from 75 to 140 K (see Fig. S7c of the SI). Lasing at 140 K is thus observed with the next available confined optical mode that has a better overlap with gain at 140 K, i.e., the TE_9,1_ mode instead of TE_10,1_ (Fig. 5c). The TE_9,1_ mode energy is above the onset of spontaneous emission (Fig. 5d). This mode switching when changing the temperature is nonetheless not systematically observed. As discussed in the SI (Fig. S7a, b), we did not observe such mode switching for 400-nm-thick disk mesas with 3 µm diameter. We can note that the TE_10,1_ mode for sample with 400-nm-thick GeSn is red-shifted by 7 meV with respect to the TE_10,1_ mode in the 360-nm-thick GeSn sample (see Fig. S8 of the SI). With this sample the TE_10,1_ mode should thus maintain a better spectral overlap with the optical gain than the TE_9,1_ mode when the temperature increases from 75 to 140 K. The slope drop at high pump intensity as observed in the inset of Fig. 5c corresponds to the roll-off of the lasers^38^, as a consequence of self-heating and modified carrier distribution spatially and in k-space.

As proof of the feasibility of tuning, in GeSnOI mesas, the laser wavelength and bandgap with external tensile strain, we also probed with pulsed excitation all-around GeSnOI mesas. After the conformal deposition of the top SiN stressor layer, an additional etching of the SiN around the MD was performed to avoid compressive strain injection at the edges of the mesas. In that case, as shown in Fig. 6a, we see a clear redshift of the emission, from 2.4 to 2.8 µm, be it below and above the lasing threshold. Such a redshift is due to the applied in-plane tensile strain of 0.5% as deduced from Raman, PL, and FEM analysis.

A more detailed analysis of the lasing performances presented in the SI (section E) however showed that all-around GeSnOI mesas did not have improved lasing performances despite a stronger lifting of the valence band degeneracy, which should have resulted in a transparency threshold reduction. The strain-induced increase of the Γ–L conduction band energy barrier was also expected to improve the maximum lasing temperature. Here, the lasing threshold of 3 µm diameter, tensile-strained GeSnOI all-around mesas was 15.5 kW/cm^2^, to be compared with 12 kW/cm^2^ for quasi-relaxed GeSnOI mesas without SiN stressors on top. The maximum lasing temperature was also slightly lower, 130 K for all-around mesas instead of 140 K without SiN stressors on top (SI section E). The optical gain was thus weaker. Tensile-strained mesas featured TM-polarized optical gain. The lasing mode at 2.8 µm of all-around GeSnOI mesas can be assigned the TM_7,1_ resonance. Since TM-polarized modes, here at a longer wavelength, have a stronger overlap with the Al metallic layer, it seems likely that the lower performances are due to higher TM losses (Fig. 6b). The *Q*-factor was of 2300 for the TM_7,1_ mode, a value significantly lower than the *Q*-factor of 12,740 we had for the TE_10,1_ laser mode of GeSnOI mesas without SiN on top. Increasing the bottom SiN thickness, in order to limit the TM field overlap with aluminum and improve the WGM *Q*-factors, should enable enhanced performances of GeSnOI all-around mesas as shown in the SI (Fig. S4). Aluminum could also be replaced by a dielectric cladding layer to avoid such optical losses. Several strategies using SiO_2_ layer as dielectric are proposed in the SI (Fig. S5). However, since SiO_2_ is a poor thermal conductor, one should use thin layers in the order of 100-nm-thick to maintain a good thermal dissipation. Despite degradation of thermal dissipation with thick SiO_2_ dielectric layers (e.g., 400 nm) as replacement of Al, the planar GeSnOI structure maintains a clear superiority as compared to the suspended GeSn-MD approach for down-scaled geometries (Fig. S5c). Aluminum was selected here as it could be etched selectively with respect to GeSn and SiN in order to fabricate suspended structures, as in ref. ^22^. We show here the interest of using a GeSnOI approach with a bottom stressor that is compatible with an all-around strain transfer scheme without suspending the active GeSn layer, thereby improving thermal dissipation while benefiting from high optical confinement. In the SI (Fig. S10), we show that, in contrast, the use of a single side SiN stressor on the top surface of a suspended MD^24,25,34^ is not suitable for homogeneous strain injection into the GeSn active layer. Such a single stressor approach indeed resulted in optical gain quenching.

MD mesas with SiN stressors have many advantages as optical laser cavities, such as scalability with the operating wavelength and high *Q*-factor provided by WGMs. One of their drawbacks, though, is the in-plane circular spread of the WGM radiation pattern. It differs markedly from the auto-collimated beam from conventional lasers, making emitted power collection tricky. The two common approaches to extract power are (i) side or top collection of radiated power with optical apparatus such as microscope objectives (ii) evanescent coupling of the disk WGM with expanded waveguide modes. The first does not yield a total collection of the in-plane radiation pattern, due to the limited numerical aperture of the collecting objectives. The second approach, with addition of some process complexity, requires an optimization of light injection into the waveguide. Critical coupling conditions have to be met by fine-tuning the gap between waveguides and MDs. Such an evanescent coupling also occurs over a limited fraction of the disk periphery and has an impact on the cavity *Q*-factor and therefore on its lasing properties. We show here that GeSnOI mesas are advantageous for a proper light collection. Indeed, circular diffraction gratings can be added around disk-shape mesas to redirect the light vertically.

**Fig. 7:**
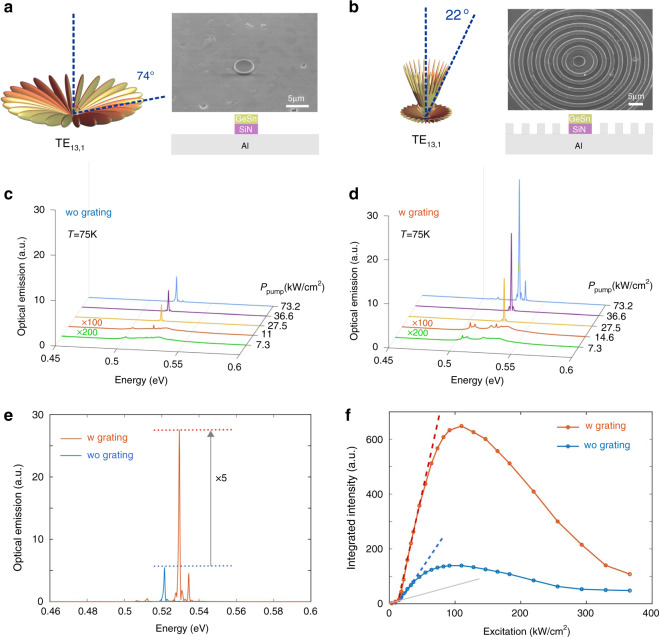
Vertical out-coupling of GeSnOI mesa lasing WGM. **a**, **b** SEM images and calculated emission patterns of 4 µm diameter GeSnOI mesas without and with Al circular grating (7 rings spaced by 3 µm with a duty cycle of 50%). Calculations have been performed with an aperiodic Fourier modal method dedicated to body-of-revolution objects^45^. **c**, **d** Power-dependent spectra without and with Al circular grating. **e** Spectra with and without grating for a 73.2 kW/cm^2^ excitation density. **f** L–L curves of GeSnOI lasers with and without grating

Here, we collect the MD emission vertically with an ×50 objective with a 0.65 numerical aperture. Light is thus collected from the sample surface within a solid angle of 40°. Yet, standard MDs have a radiation pattern which is mainly out of this solid angle, as shown in Fig. 7a, with a modeling of the WGM radiation for a 4 µm diameter GeSnOI mesa MD. A small MD diameter was chosen as it simplified the modal analysis. Such a mesa features a monomode lasing at 0.52 eV which can be assigned to the TE_13,1_ mode at an energy of 0.54 eV. The maximum radiation angle is situated around 74° with respect to the normal incidence (Fig. 7a). These simulations also show an extremely small coupling factor from such WGM pattern to the collecting objective, of the order of 10^−5^ only. The collected emission from WGM lasing therefore comes from out-of-plane leakage rather than from radiative emission. Such leakage paths stem from imperfection introduced during fabrication (sidewall and surface roughness, circular symmetry breaking). These imperfections, useful to collect more light into the collection angle, are obviously unwanted in practice since they result in lower *Q*-factors and increase lasing thresholds.

One way of overcoming this issue would be to have an optimized process flow in order to reach the highest *Q*-factors possible and use external gratings to redirect the WGM in-plane radiation pattern. This is feasible with GeSnOI mesas, while it would be very tricky with GeSn suspended MDs. We thus designed a circular diffraction grating with optimal period and duty cycle to maximize the redirection of the WGMs light into the 40° solid angle for a 4 µm diameter GeSnOI mesa (see the SI). Figure 7b radiation diagram with such additional circular grating is drastically modified for the TE_13,1_ mode, with then a maximal radiation angle of 22° compared to normal incidence. The total flux integrated into our objective collection angle becomes theoretically of 30% as compared to 2.4 × 10^−5^ without grating. Such a power collection improvement is expected for lasing WGM mode specifically coupled to the grating. The GeSnOI mesa laser emission spectra and integrated collected intensity, below and above laser threshold, are shown in Fig. 7c–f with and without grating. Below the threshold, spontaneous emission dominates the spectrum and the contribution from WGM is weak as it is not amplified. Intensities with and without grating are thus equivalent. On the contrary, above a threshold which was around 15 kW/cm^2^ in both cases, the WGM contribution dominates the spectrum and a clear improvement of collected intensity is observed, typically by a factor of 5 for mesas with gratings. The maximum collected peak power is around 20–30 µW.

The modeling predicted an improvement by 12,500 of the intensity collected by the objective with such a grating. It is thus very clear that initially, the collected light without the grating mainly stems from leak paths, as mentioned above, due to roughness scattering not accounted for in the modeling. The very weak estimated coupling factor of the WGM radiation to the objective, of the order of 10^−5^ for mesa MDs, is thus not representative of the power collected experimentally. Such scattering obviously tends to reduce the cavity quality factor of the TE_13,1_ mode calculated to be *Q*_rad_ = 8160 for perfectly smooth surfaces and sidewalls. Such a method would thus yield a better control of the total power radiated from the cavity without changing the cavity geometry and its lasing characteristics. The same emission control is feasible for larger diameter cavities. The 4 µm diameter featuring single-mode lasing was chosen here to simplify the experimental analysis, but similar results with higher diameter disks have been simulated (data not shown). Gratings without aluminum could as well be envisaged.

## Discussion

On the basis of our proposed GeSnOI platform, one could also obtain in-plane coupling of radiated emission into waveguides if the aim was to inject the laser emission into a photonic circuit. The most obvious way would be to use SiN, which offers a high optical index and high transparency in the mid-infrared, as the waveguide material. SiN was indeed proven to be suitable for mid-IR photonic circuits in the literature^39,40^ and could be combined with GeSn as active materials. This should be feasible with our proposed GeSnOI stack. Selective deposition of SiN could be used if an increase in the waveguide core thickness in some places was needed. Blanket SiN deposition followed by selective etching of some of it in specific places, as used here to fabricate all-around mesas, could be selected to construct the in-plane photonic circuit. SiN etching can for instance be performed using fluoride-based plasma, which is very selective to GeSn^41^. The aluminum layer might have to be replaced by low index dielectrics cladding such as silicon dioxide, then^42^. As discussed above and detailed in the SI (section C), the replacement of the Al layer requires specific designs with the aim to maintain a good thermal dissipation for the laser devices. We note that the mesa structure offers superior thermal dissipation than under-etched GeSn/Ge SRB layer while having equivalent optical confinement factors. In this work, we used Al grating for light beam engineering with GeSnOI. There are many others possibilities, using dielectric instead of Al material for that purpose when using infrared compatible materials. In the latter cases, the periodicity and the dimension will depend on the optical parameters of the chosen material. We can further envision more complex mode radiation shaping as compared to the one provided in the present work, by using phase engineering of the wave fronts.

We have shown here the interest of using GeSn active layers bonded on dielectric stressors to fabricate GeSnOI stacks, resulting in optimized group-IV laser designs in terms of defects, strain, modal, and thermal engineering. Additionally, bandgap energy and directness parameters could be tuned via tensile strain. The strain can be varied by using different parameters for the SiN stressor deposition, by varying the Sn content of the alloy, and the degree of relaxation of the GeSn layer through the conditions of epitaxial growth. Obviously, such an approach can be applied to different GeSn active layers with varying Sn contents, as well as complex heterostructures with quantum wells, to provide additional electronic band and gain engineering. The first purpose of the present work was to show the relevance of GeSnOI MD mesas compared to more conventional structures such as suspended GeSn MDs on Ge pillars. The same as-grown 500-nm-thick GeSn 10.5% layer was used for such a comparison. GeSnOI mesas have approximately the same high index contrast than suspended MDs and are suitable for the fabrication of planar structures, free from interface defects, such as ridge Fabry–Perot waveguides, ring cavities, or even photonic crystals. Enhanced performances were obtained with the GeSnOI approach as compared to conventional approaches. We highlighted that planar emission can be redirected vertically thanks to additional gratings, illustrating the flexibility provided by such a technology.

The key advantage of this GeSnOI platform is its ability to combine active laser structures with passive SiN circuitry from the near-infrared to the mid-infrared. It represents a new paradigm for infrared Group-IV photonics that eliminates the need for III–V laser integration.

To be able to reach temperature with this GeSnOI approach, one of the most obvious way is to increase the tin content of the alloy. Here we have reported a complete study as a first proof of concept to assess the GeSnOI laser technology with respect to the conventional GeSn/Ge SRB, starting from a GeSn with a 10.5% tin content. It has been shown in the literature that lasing up to 270 K can be reached in suspended structures, thus with poor thermal management, using under-etched GeSn/Ge SRB cavities^32^. In this case, a tin content of 16% in the active region was used. The increase of tin content induces an increase of the directness of the band structure that is one of the main key ingredient which allowed for the higher-temperature laser operation as compared with lower tin content materials. As the GeSnOI approach exhibits improved performances, we can thus expect that this approach, if applied to materials with higher tin content than in this work, could lead to even higher temperatures of laser operation.

One second challenging goal is to demonstrate electrically driven devices. We emphasize that this is completely compatible with our approach if we start with GeSn pin junctions since the bonding can be applied to any kind of GeSn heterostructures. Bonding GeSn diodes is completely feasible and the electrical injection can be made with such GeSnOI active layers. The GeSnOI can even offer better performances as compared to a standard approach since the high index contrast offers more flexibilities in the design to reduce the overlap of the laser modes with doped contacts and the electrodes. It has been shown that electrical injection in optical microcavities is compatible with the use of the SiN stressor transfer method, as long as specific designs are used to define the electrode geometries^43^.

## Materials and methods

### GeSn on Ge SRB material growth on silicon

Epitaxial growth of close to 500-nm-thick GeSn 10.5% was performed in a 200 mm Epi Centura 5200 cluster tool from Applied Materials on a Ge SRB on silicon. The Ge SRB was grown with GeH_4_ and a low temperature/high temperature approach, followed by some short duration Thermal cycling to reduce the threading dislocations densities to values around 10^7^ cm^−2^, typically. Meanwhile, the GeSn layer was grown close to 337 °C at 100 Torr thanks to Ge_2_H_6_ and SnCl_4_ precursors^44^. The as-grown layers was analyzed by X-ray diffraction reciprocal space mapping. The layer has a residual compressive strain of −0.5%.

### WGM radiation modeling

The WGM analysis and their scattering by the Al grating was performed in the framework of the aperiodic Fourier modal method that was detailed in ref. ^45^. The permittivity model considered, here and in previous sections, for aluminum was a realistic Lorentz–Drude model^46^. More details are provided in the SI (section G) for the design of the Al grating.

## Supplementary information

Supplementary Information-GeSnOI mid-infrared laser technology
