# Supplementary material for: GeSnOI mid-infrared laser technology
Source: Light Sci Appl. 2021 Nov 17;10:232. doi: 10.1038/s41377-021-00675-7 (PMC8595695; doi:10.1038/s41377-021-00675-7)
Supplement: Supplementary file 1 — Supplementary Information-GeSnOI mid-infrared laser technology [file 41377_2021_675_MOESM1_ESM.docx]

Supporting information

**GeSnOI mid-infrared laser technology**

Binbin Wang,^1^ Emilie Sakat,^1^ Etienne Herth,^1^ Maksym Gromovyi,^1^ Andjelika Bjelajac,^1^ Julien Chaste,^1^ Gilles Patriarche,^1^ Philippe Boucaud,^2^ Frédéric Boeuf,^3^ Nicolas Pauc,^4^ Vincent Calvo,^4^ Jérémie Chrétien,^4^ Marvin  Frauenrath,^5^ Alexei Chelnokov,^5^

Vincent Reboud,^5^ Jean-Michel Hartmann,^5^ Moustafa El Kurdi^1,∗^

*1 - Université Paris-Saclay, CNRS, C2N, 10 boulevard Thomas Gobert, 91120, Palaiseau, France*

*2- Université Côte d’Azur, CNRS, CRHEA, Rue Bernard Grégory, 06905 Sophia-Antipolis, France*

*3-STMicroelectronics, Rue Jean Monnet 38054 Crolles, France*

*4-Université Grenoble Alpes, CEA, IRIG-DePhy, 17 rue des Martyrs,38000 Grenoble, France*

*5- Université Grenoble Alpes, CEA, Leti, 17 rue des Martyrs, 38000 Grenoble, France*

**Content :**

**A-Fabrication of GeSnOI disk mesas**

**B-Optical confinement**

**C-Thermal analysis**

**D-Laser characterizations**

**E-Laser performances of all-around GeSnOI mesas**

**F-Tensile strain injection in GeSn microdisks**

**G-WGM analysis, grating parameter optimization**

**A-Fabrication of GeSnOI microdisk mesas**

Processes started from a 500 nm thick GeSn layer with 10.5% of tin grown on a 2.5μm thick Ge strain-relaxed-buffer (Ge-SRB), itself on a 200mm Si(001) wafer. The same layer was used in Ref. [1], with detailed structural data provided. A 400 nm thick silicon nitride (SiN) layer was firstly deposited on the top surface by plasma-enhanced chemical vapor deposition (PECVD). An 800 nm thick Al layer was then deposited by e-beam evaporation. Then, another 50 nm thick SiN layer was deposited by PECVD prior to Au deposition. It was used as a diffusion barrier between Al and Au layers. The 400 nm thick Au layer was finally deposited for bonding. The 400 nm thick Au layer was also deposited on a host Si substrate at this step. The two samples were then bonded using Au-Au thermo-compressive bonding at 300 °C. After bonding, the Si substrate on which the GeSn layer was grown was thinned down to a thickness around 50 μm with mechanical polishing. The remaining Si substrate was removed thanks to a KOH wet etching which was selective over Ge. The 2.5 μm thick Ge SRB was then removed with a sulfur hexafluoride (SF_6_) based deep reactive ion etching (DRIE). At this step, the defective GeSn layer at the interface between GeSn and the Ge SRB layer was also removed. The etching depth was controllable because of the moderate etching rate of GeSn over Ge. In this work, we typically obtained GeSn thicknesses between 360 nm and 400 nm as obtained from different batch process. At this stage, an improved GeSn layer on SiN, free from interface defects, was obtained, thus leading to the GeSnOI stack. Fig. S1 shows a zoomed cross-section TEM image of the as-grown GeSn layer on the Ge-SRB, which shows a dense array of misfit defects near the interface (interface defects). A small portion of the defects extend above the interface and the process induces a surface roughness. After bonding to form the GeSnOI stack, the dense misfit defects array near the GeSn/Ge-SRB interface is removed. We estimate [2] that the density of the GeSn/Ge interface defects is of the order of 10^11^ cm^-2^ while the density of the extended defects is 3 order of magnitudes lower, i.e. 10^8^ cm^-2^.


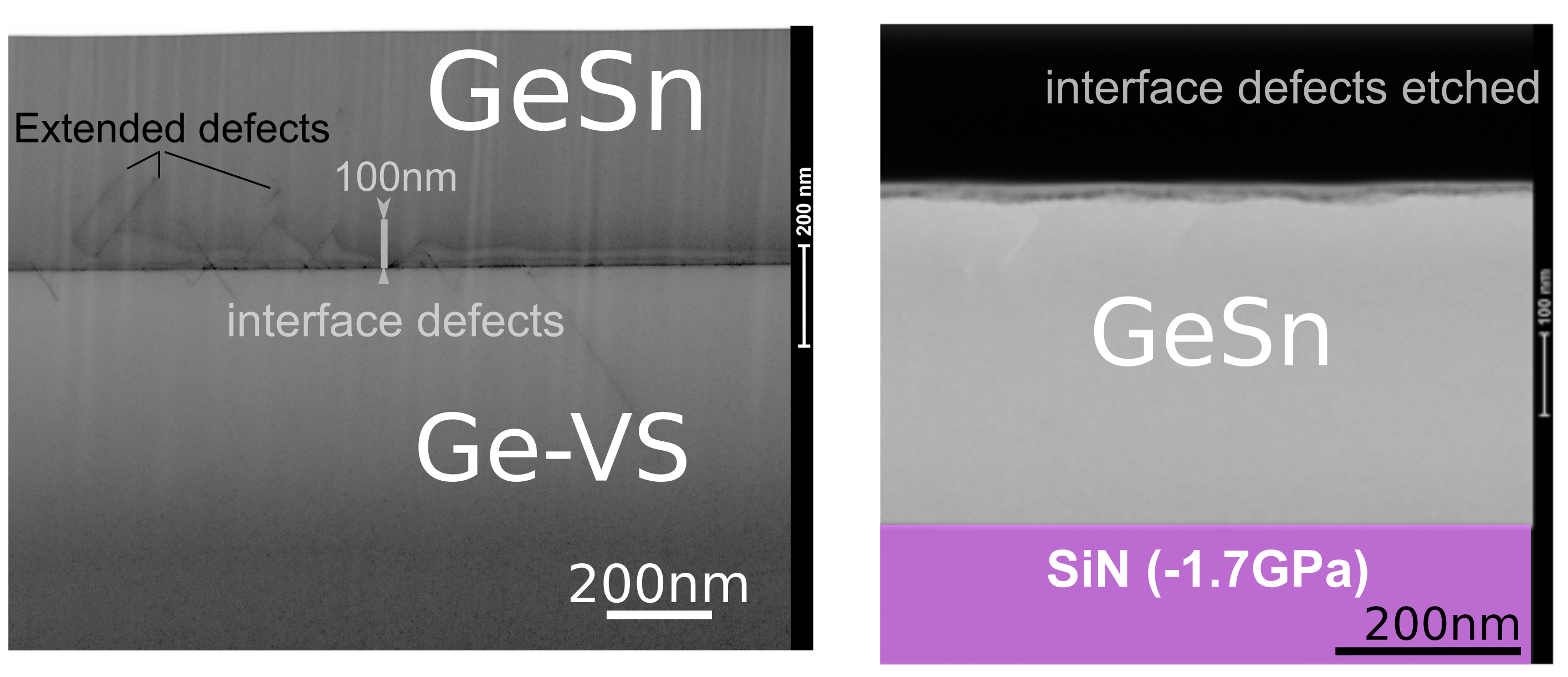


Figure S1 X-TEM image of the as-grown layer (left) and of the GeSnOI layer obtained after bonding. Most of the defects near the GeSn/Ge SRB interface were etched from the GeSnOI layer. The top surface has roughness of the order of 10 nm height.

Afterwards, the GeSnOI blanket structure was patterned, using standard e-beam lithography and etching to define the microdisk mesa resonators, as shown in Figure S2a.

In the future, one could replace the Au-Au bonding with a cost-effective BCB (Benzocyclobutene) bonding, which was found to be feasible for the bonding of GeSn layers, as shown in the cross-section image of Fig. S2b.

| 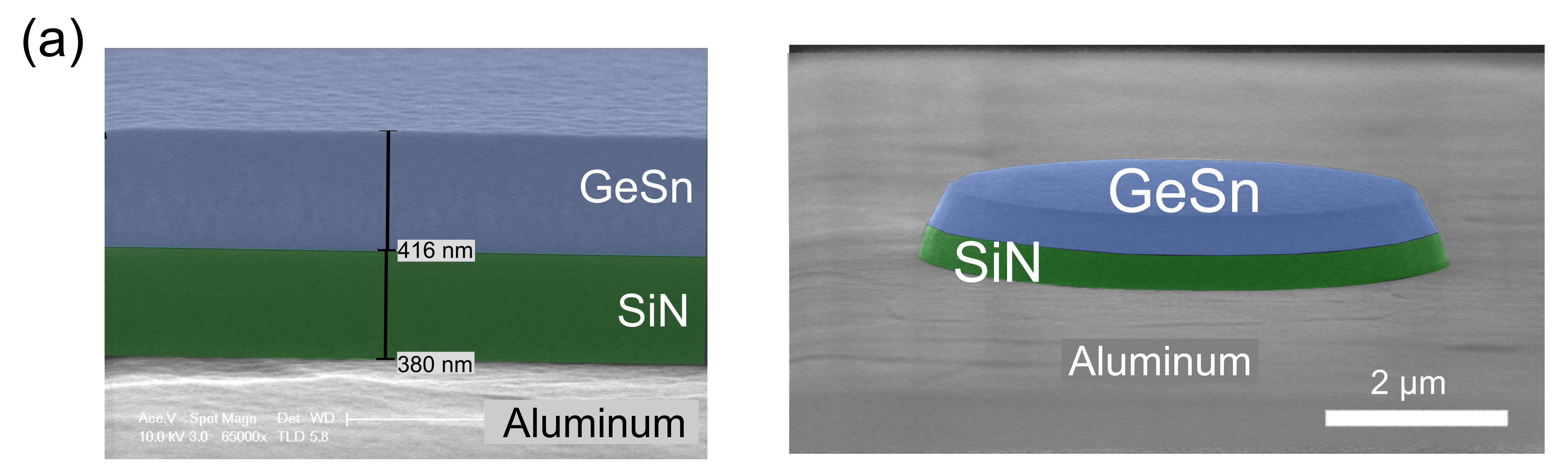    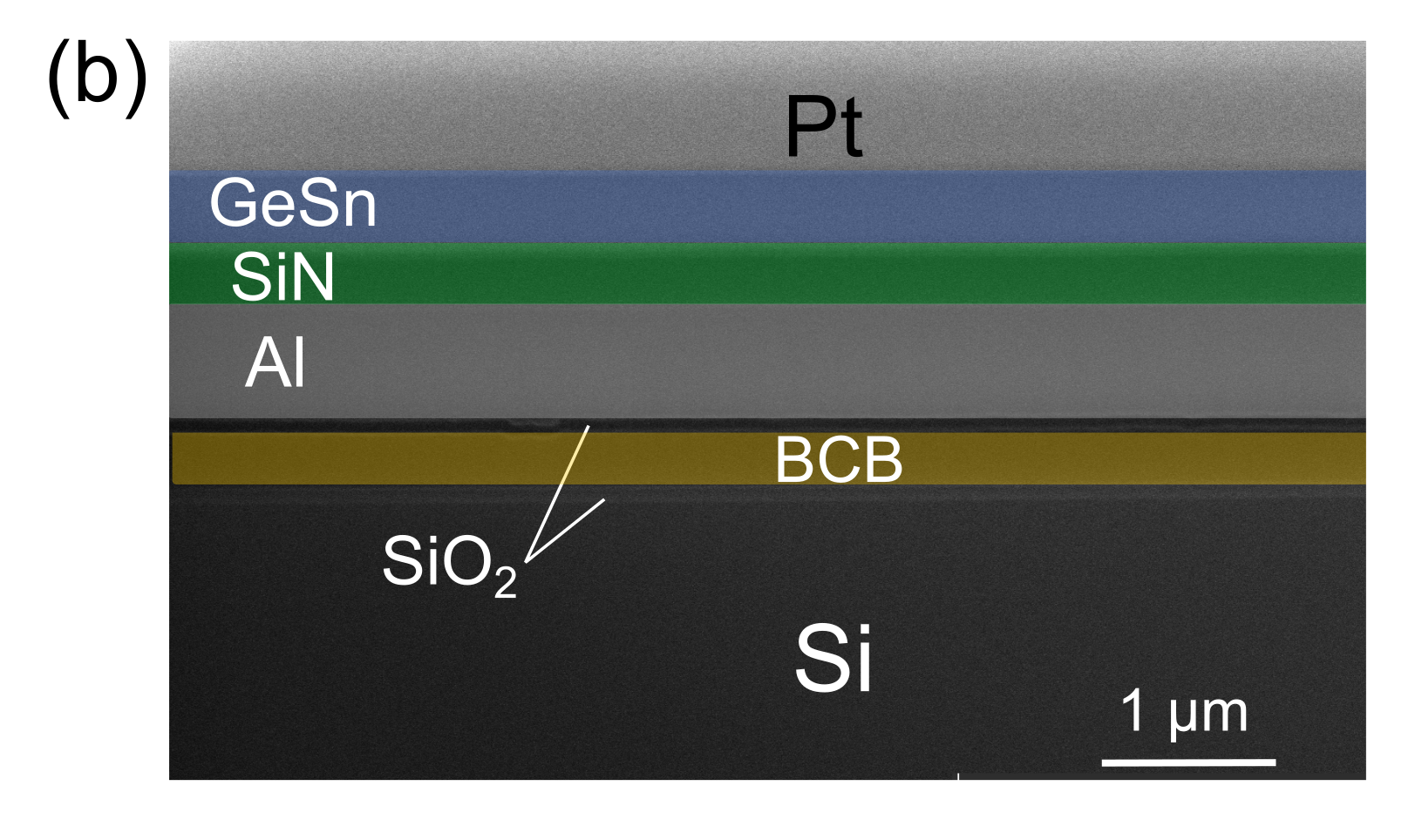 |
| --- |

Figure S2. (a) SEM images of the GeSnOI stack and a resonator microdisk mesa. (b) Cross-section SEM image obtained after Focused Ion beam (FIB) etching of the bonded layer, with a protective ion beam melted Pt cap. The GeSnOI stack was obtained using a cost-effective Benzocyclobutene bonding method instead of Au-Au bonding.

**B-Optical confinement**

Because of a weak refractive index contrast between GeSn and Ge SRB in the 2-3 µm wavelength range  [3], the as-grown GeSn layer on Ge-buffered silicon has very weak confinements for TE and TM polarizations (Figures S3a and S3c), while the GeSnOI stack clearly has better confinement for TE and TM polarizations (Figures S3b an S3d).

**
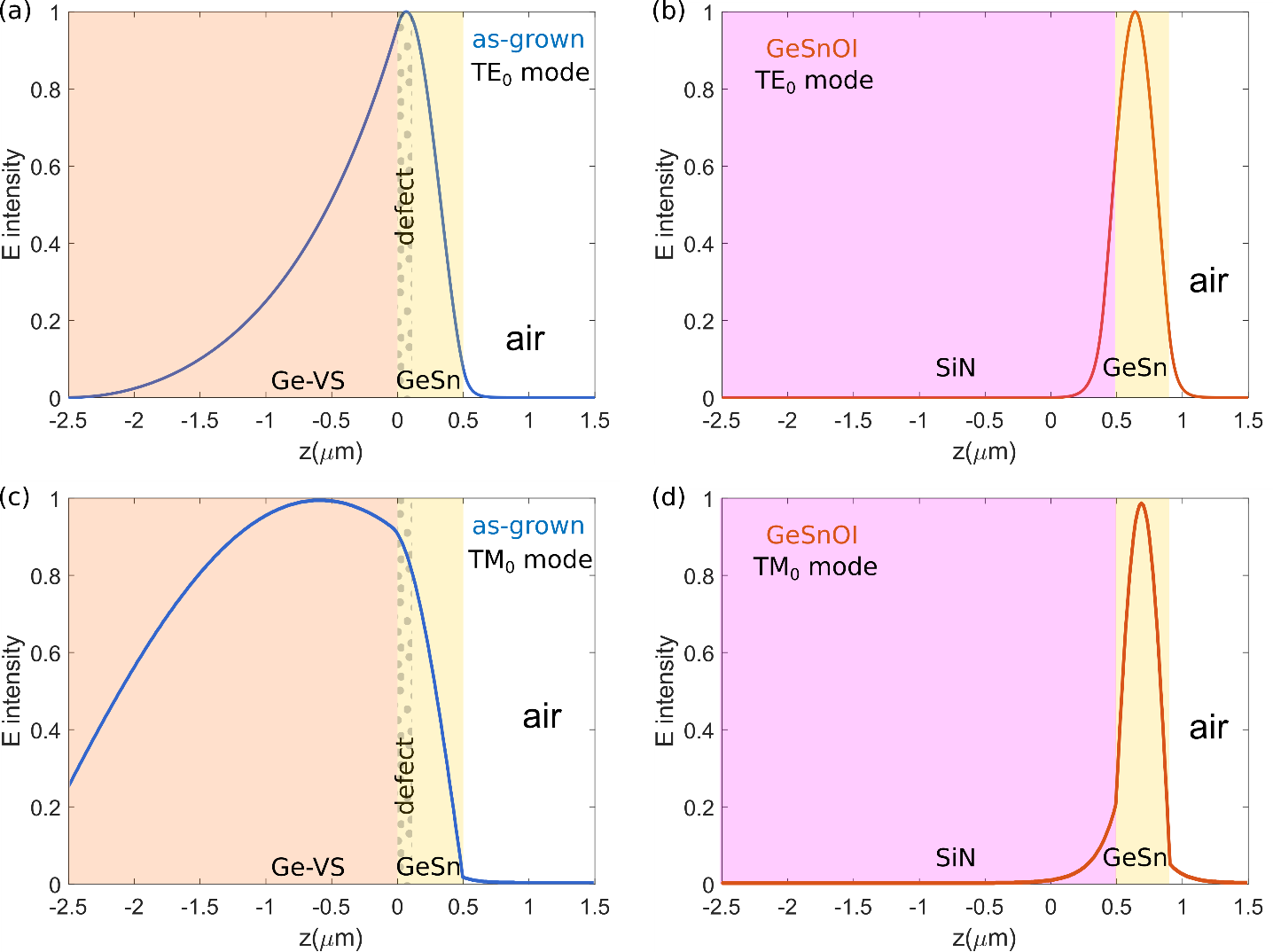
**

Figure S3. Electric field intensity of TE_0_ mode at 2.4 µm wavelength of (a) of the as-grown GeSn layer on a 2.5 microns thick Ge SRB, itself on a Si substrate (b) a GeSn layer on silicon nitride. (c) and (d) are the same as (a) and (b) but for the TM_0_ mode.

The use of an Al layer may introduce undesired metallic losses. This is avoided by using a thick enough SiN insulator layer. A 350 nm thick SiN layer is enough to keep metallic losses below $1\times{10}^{-3}dB.{\mu m}^{-1}$ (Fig. S4). Thicker layers would be preferable to reach high Q factors for TM-polarized whispering gallery modes, as discussed in the main text.


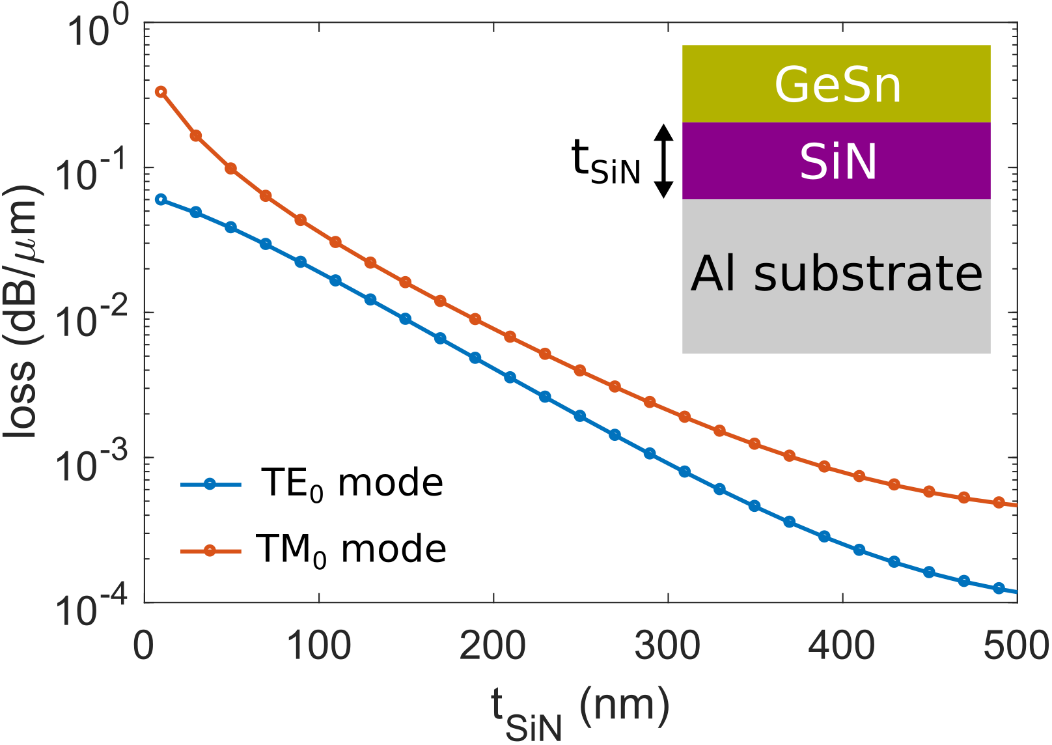


Figure S4. Impact of SiN layer thickness on losses at a 2.4 μm wavelength. Simulated losses of fundamental TE mode and fundamental TM mode of 1D GeSn-SiN-Al stacking. GeSn and Al thicknesses in those simulations were 500 nm and 1μm, respectively.

**C-Thermal analysis**

To investigate the excitation source - induced heating of GeSn microdisk lasers, we performed local thermal dissipation simulations, with a COMSOL Multiphysics software, for 7 μm diameter GeSn microdisks and GeSnOI mesas. Results are shown in Fig. S5. Temperature-dependent thermal conductivities of Ge, Si, Al, and Au were coming from Ref.  [4] and  [5]. The excitation source was modeled as a homogeneous pulsed 2D heat source with a spot diameter of 12 μm. A reflectivity of 0.35 was taken into account due to the surface reflection of the 1550 nm wavelength pump laser on the GeSn surface.

As shown in Fig. S5a, a 200 kW.cm^-2^ pump power resulted in a 15 K increase of the temperature in GeSn microdisks with 7 µm diameters and pedestals 3 µm wide only. By contrast, the maximal temperature of GeSnOI mesas almost did not change because of the good heat dissipation with such stacks. The average temperature within the active region linearly increased with the excitation density in both configurations (Fig. S5c). The temperature in the microdisk was otherwise higher than at its periphery than at its center (Fig. S5a). This was a clear signature of a thermal flux from the edge to the center of GeSn microdisks, where the Ge pedestal provided the thermal connection to the Si substrate sink. The temperature of GeSn microdisks otherwise increased exponentially when the microdisk diameters became smaller than 6 μm (for fixed 2 µm undercuts). In the latter case, this corresponded to pedestal diameters smaller than 2 µm. This was the likely reason why GeSn microdisks did not lase for disk diameters smaller than 5 μm. On the contrary, GeSnOI mesas benefited from thermal dissipation even for very small microdisk diameters, enabling the analysis of laser characteristics for mesas as small as 3 µm in diameter. One can replace the Al layer by a dielectric material in order to address the optical loss issues. We have extended the modeling to the following cases: the aluminum layer is replaced by a SiO_2_ layer with thickness of 400nm or 100 nm; the aluminum layer is replaced by a SiN layer that exhibits a much better thermal conductivity as compared to SiO_2_.


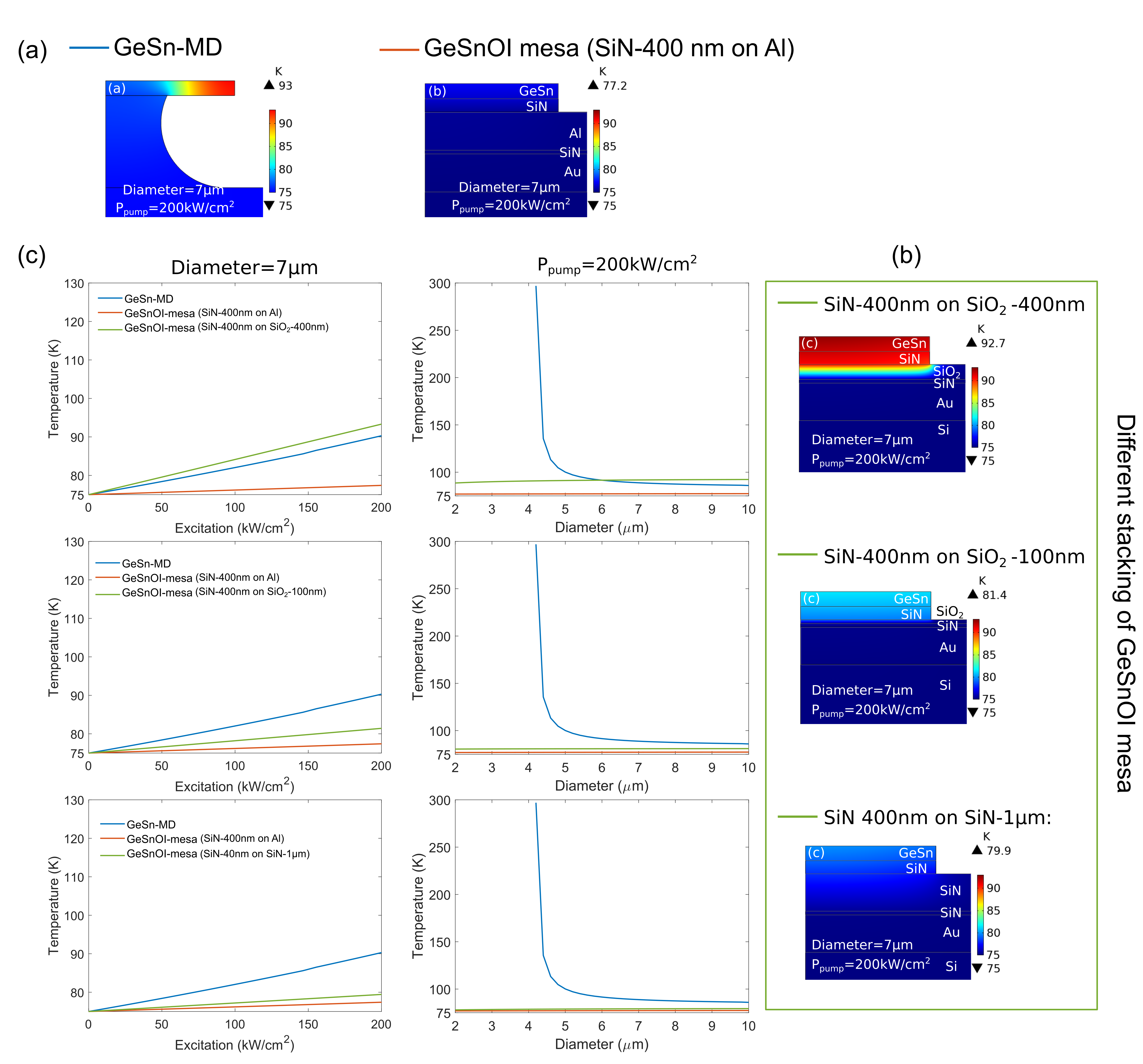


Figure S5. (a) Simulated 2D temperature distribution in GeSn microdisks and GeSnOI mesas at a pulsed excitation density of 200 kW.cm^-2^ and a duty cycle of 0.1. The microdisk or mesa diameter was 7 μm and the substrate temperature set as 75 K. The microdisk under-etching is 2 μm. (b) Various schemes of GeSnOI stacks where the Al layer is replaced by dielectric materials, from top to bottom: 400 nm thick SiO_2_, 100nm thick SiO_2_ and 1µm thick SiN. The 2D temperature distributions in these different GeSnOI mesas with identical diameter of 7 µm as in (a) and under the same pumping condition as in (a) are given by the color bars. (c)-Left, temperature in the active region as a function of excitation density for 7 µm diameter GeSn microdisks (blue line) and GeSnOI mesas with the Al layer (red line) and with replacing the Al layer with a dielectric layer (green line) (c)-Right, temperature in the active region of GeSn microdisks and GeSnOI mesas under 200 kW.cm^-2^ excitation density for various diameters. The same colour legend as for the figure on the left was applied.

This modeling indicates that even without aluminum, one can obtain a reasonable thermal dissipation even if it remains less efficient. Replacing aluminum might decrease the optical losses associated with the metal. The mesa structures are less impacted by the presence of dielectrics as compared to mushroom-type microdisks. Different strategies are thus available to combine strain engineering and thermal dissipation. The approach proposed in this manuscript with aluminum is not the only one available.

For 7 μm diameter microdisks, the narrower roll-over in lasing performances for GeSn microdisks as compared to GeSnOI mesas (Fig. S6) was due to lower thermal dissipation and less robust gain. Because of a lower gain robustness as the temperature increased, GeSn microdisks had a quenched laser operation above 200 kW.cm^-2^ excitation and therefore a temperature that increased, because of local heating, by 15 K, i.e. a 90 K temperature only 10 K above the 80 K maximum lasing temperature we obtained from temperature-dependent analysis of lasing characteristics.

~~
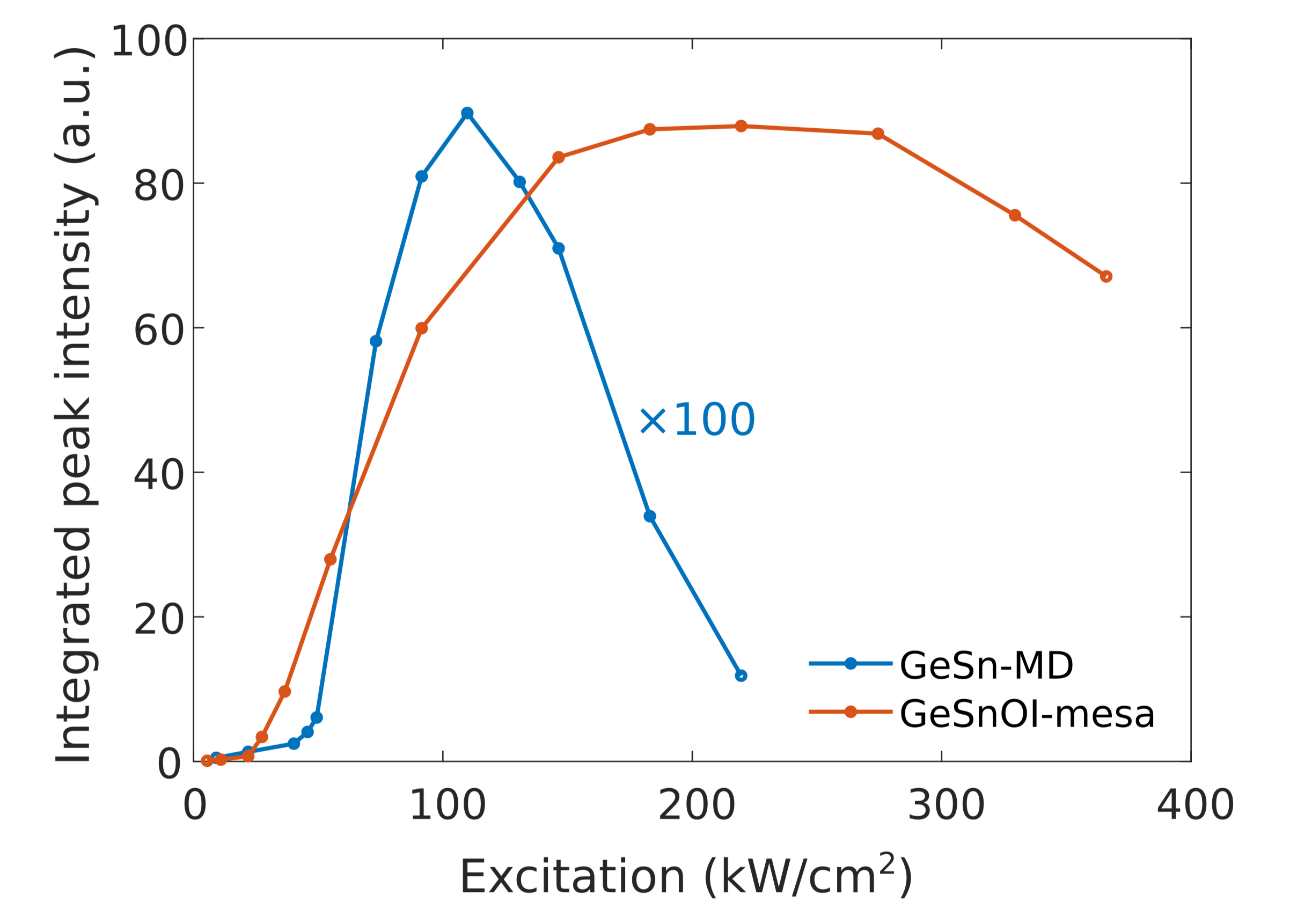
~~

Figure S6. L-L curves of GeSn microdisks and GeSnOI mesas under pulsed excitation at 75 K. The L-L curves are obtained by integrating the laser peak emission under different excitation densities. The diameters of the GeSn microdisk and GeSnOI mesas are 7 μm.

**D-Laser characterizations**

Figures S7a and S7b show a comparison of the lasing spectra for a 400 nm thick GeSn active layer and for a 3 µm diameter mesa structure. Lasing is observed on the same TE_10,1_ mode at 75 K and 140 K. The temperature increase did not induce a redshift of the optical mode which remains at 0.518 eV. This is different from Figure 5 of the manuscript where lasing was observed on a lower-energy whispering gallery mode at 140 K, as shown in Fig. S7c plotted in Log Scale.

| 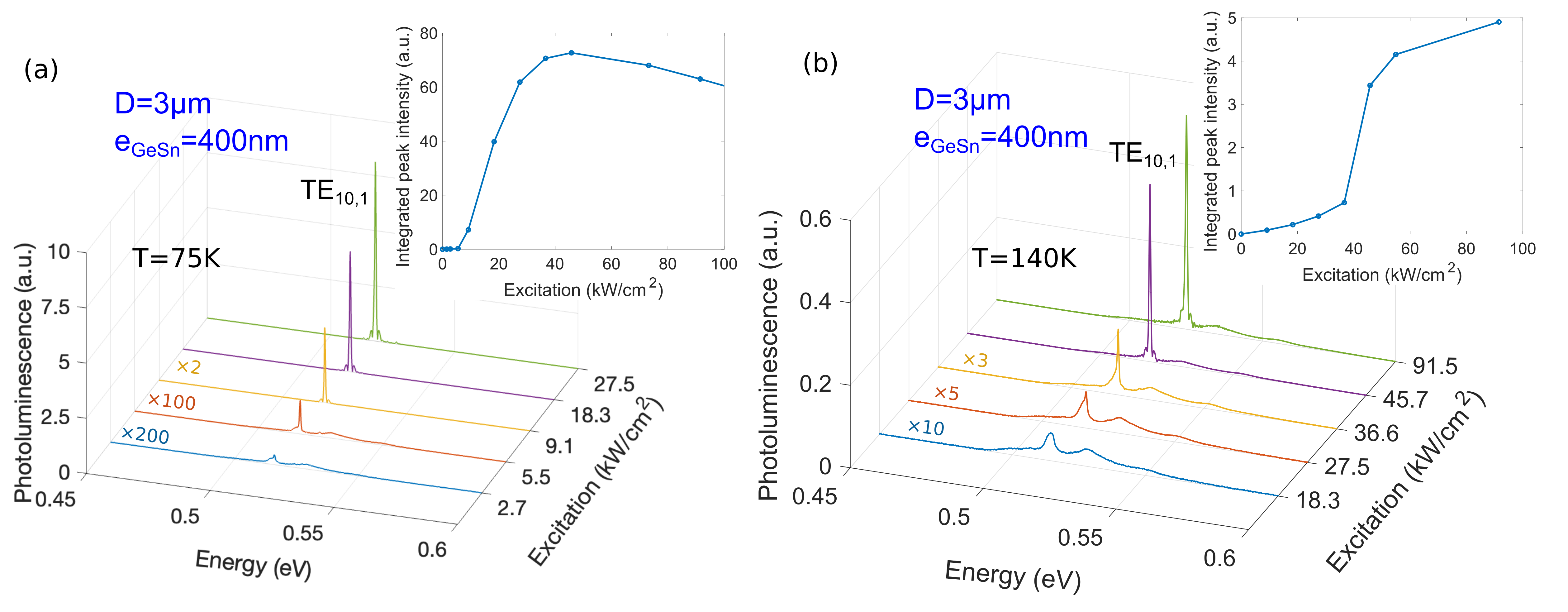 |
| --- |
| 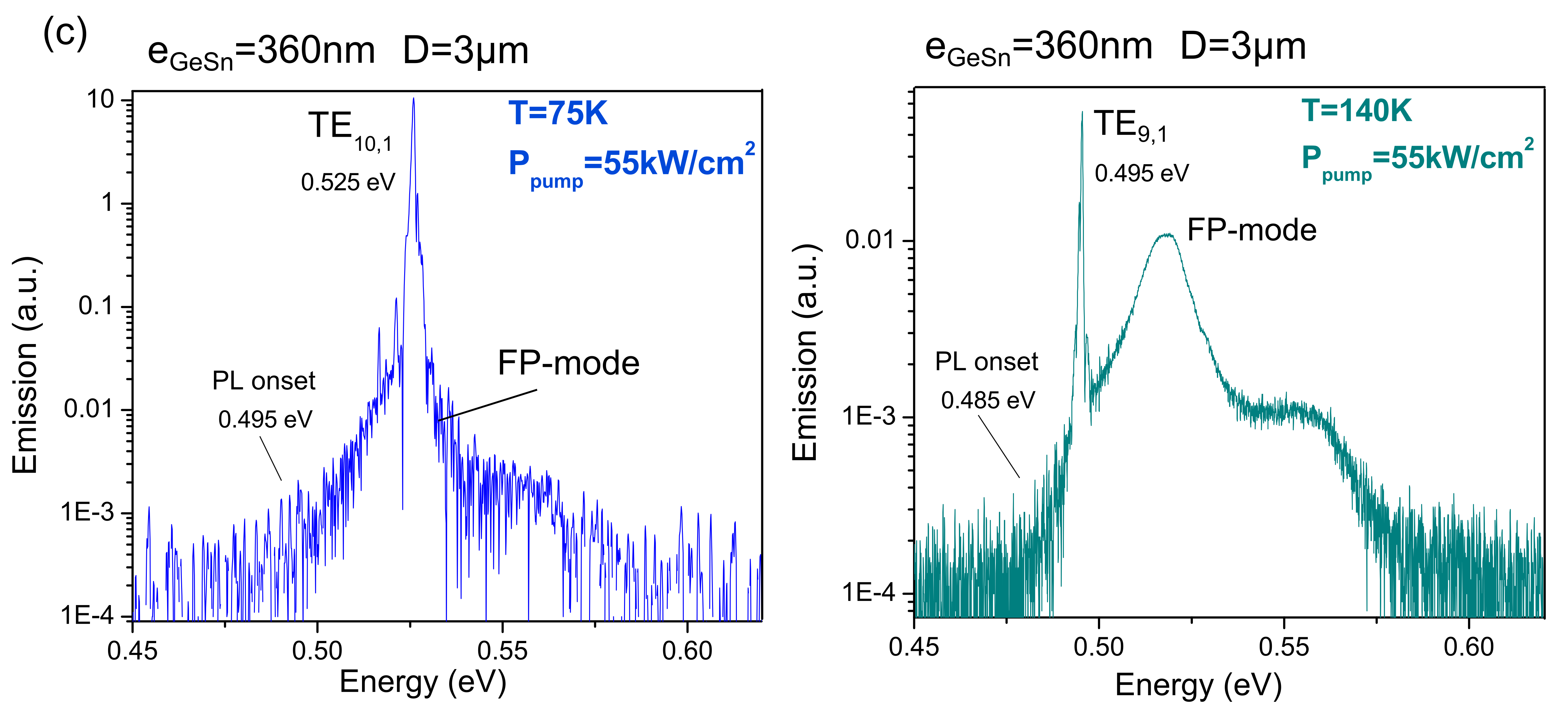 |

Figure S7. (a) Laser spectra at 75 K under different pumping power densities of a 3 µm diameter microdisk mesa that was fabricated from a GeSnOI sample with GeSn thickness of 400 nm. The inset shows the L-L curve of the lasing TE_10,1_ mode (b) same as (a) but at 140 K, the same lasing TE_10,1_ mode remains. (c) Log plot of the emission spectrum above laser threshold for 3µm diameter microdisk mesas fabricated from GeSnOI layer with 360 nm GeSn thickness (same structure shown in the figure 5 of the manuscript) at 75 K left and 140 K (right). Lasing is obtained with the TE_10,1_ mode at 75 K but with the TE_9,1_ mode at 140 K in this case.


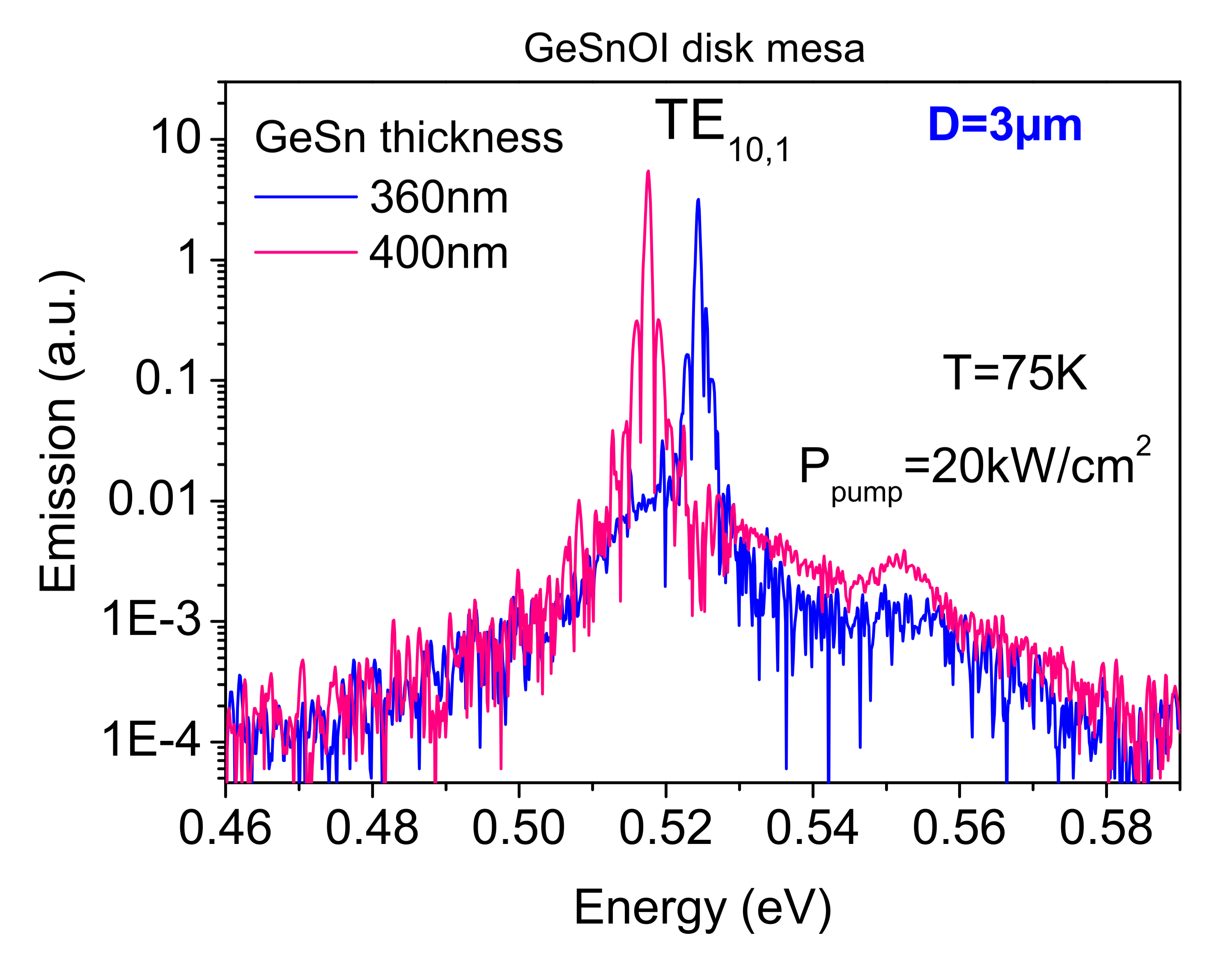


Figure S8. Comparison of the lasing spectra for microdisk mesas with different active layer thicknesses (360 and 400 nm). The lasing mode shifts by 7 meV when decreasing the active layer thickness.

Figure S8 shows a comparison of the spectral position of the whispering gallery lasing modes as a function of the thickness of the active layer (360 nm and 400 nm). The measurements are performed at 75 K under 20 kW.cm^-2^ pumping power. One observes a shift of 7 meV when increasing the thickness in good agreement with the calculated shift (11 meV) for whispering gallery modes. This is another indication that corroborates the whispering gallery origin of the lasing modes.

**E-Laser performances of all-around GeSnOI-mesas**

In Figure 6 of the main text, we only show the laser spectral shift between 3 μm diameter GeSnOI mesas and all-around GeSnOI mesas under an excitation density of 55 kW.cm^-2^. In Fig. S9 we show the full laser performances of 3 μm diameter all-around GeSnOI mesas. At 75 K, the threshold density is 15 kW.cm^-2^ and the maximal laser intensity is obtained at 55 kW.cm^-2^ (Fig S9a), both of them lower than for 3 μm diameter GeSnOI mesas as in Fig. 5a. Lasing is observed up to 125-130 K (Fig S9b). The temperature-dependent spectra (Fig. S9c) and L-L curves (Fig. S9d) clearly confirm a maximal lasing temperature of 130 K, also lower than the maximal lasing temperature of the 3 μm GeSnOI microdisk without the top SiN in Fig. 5a. The degraded laser performance can be attributed to larger TM-mode- overlap with the Al layer, as discussed in the main text. Note that tensile strain promotes TM-polarized optical gain.


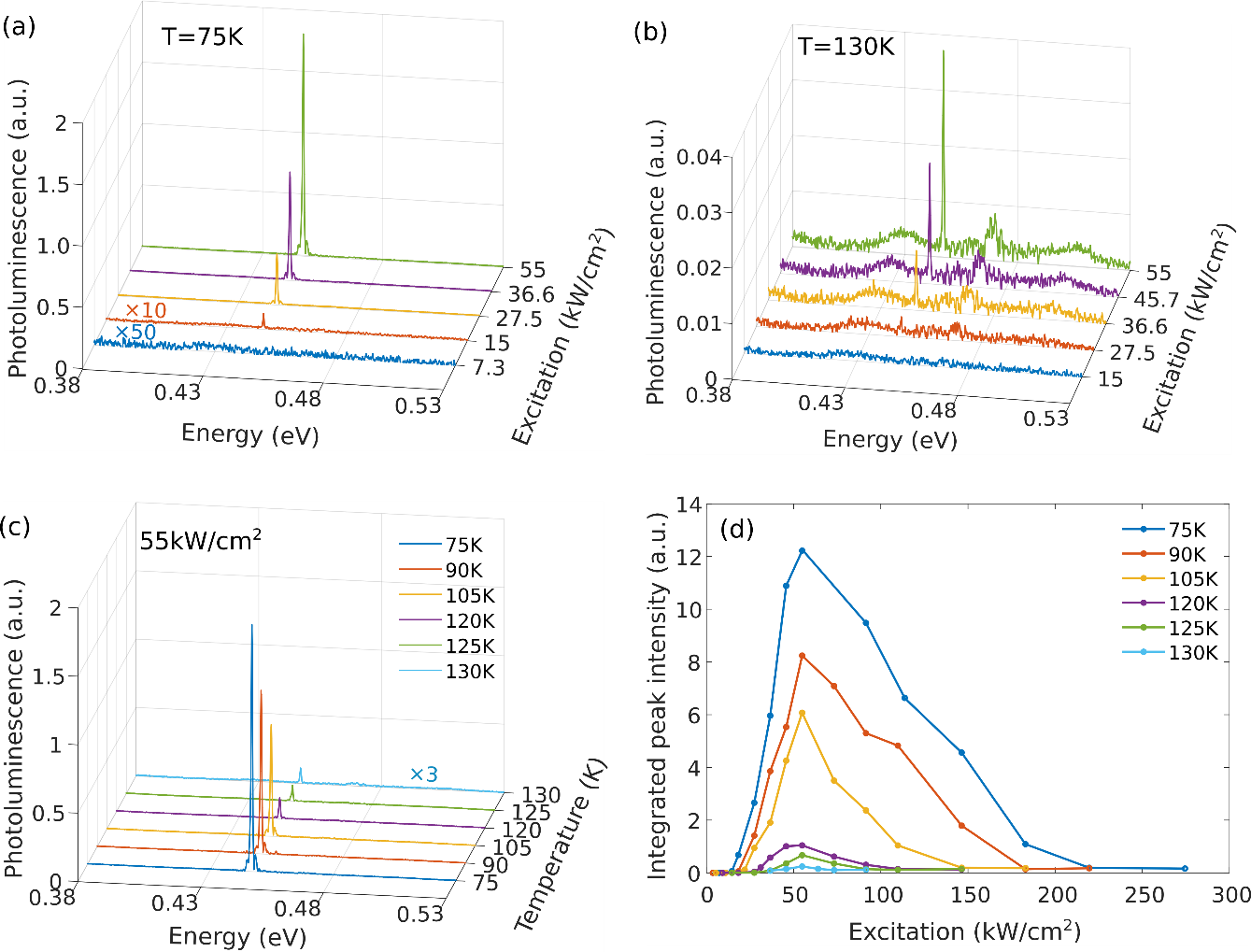


Figure S9. Laser performances of 3 μm diameter all-around GeSnOI mesas. (a) and (b) are emission spectra under various excitation densities at 75 K and 130 K, respectively. (c) shows the spectra under 55 kW.cm^-2^ excitation density at different temperatures. (d) L-L curve taken from laser peaks integral at different temperatures.

**F-Tensile strain injection in GeSn microdisks**

One would obviously like to have strain injection using a approach as straight forward and simple as possible, for instance by depositing a SiN stressor layer on top of a suspended GeSn microdisk, using thus a single side stressor layer on the top surface, as in Ref. [6]. For the sake of completeness, we show results obtained using such approach. There is in Figure S10a a significant red-shift of the PL emission after SiN deposition on the microdisk. FEM analysis of such structure predicts an in-plane transferred tensile strain close to 1% near the SiN/GeSn interface. In that case, the injected strain is highly inhomogeneous along the GeSn layer thickness (Figures S10b and S10c). The strain is the highest close to the SiN/GeSn interface. It decreases very rapidly in the bulk of the GeSn layer to become compressive on the bottom side of the free-standing microdisk. This inhomogeneous strain distribution led to modal gain quenching. We were indeed not able to have lasing despite a very high tensile strain in the GeSn layer. Meanwhile, strain-free GeSn microdisks lased (Fig. S10a). The impact of the inhomogeneous strain distribution in the microdisk layer on the optical gain was discussed in details in Ref.  [7].

**
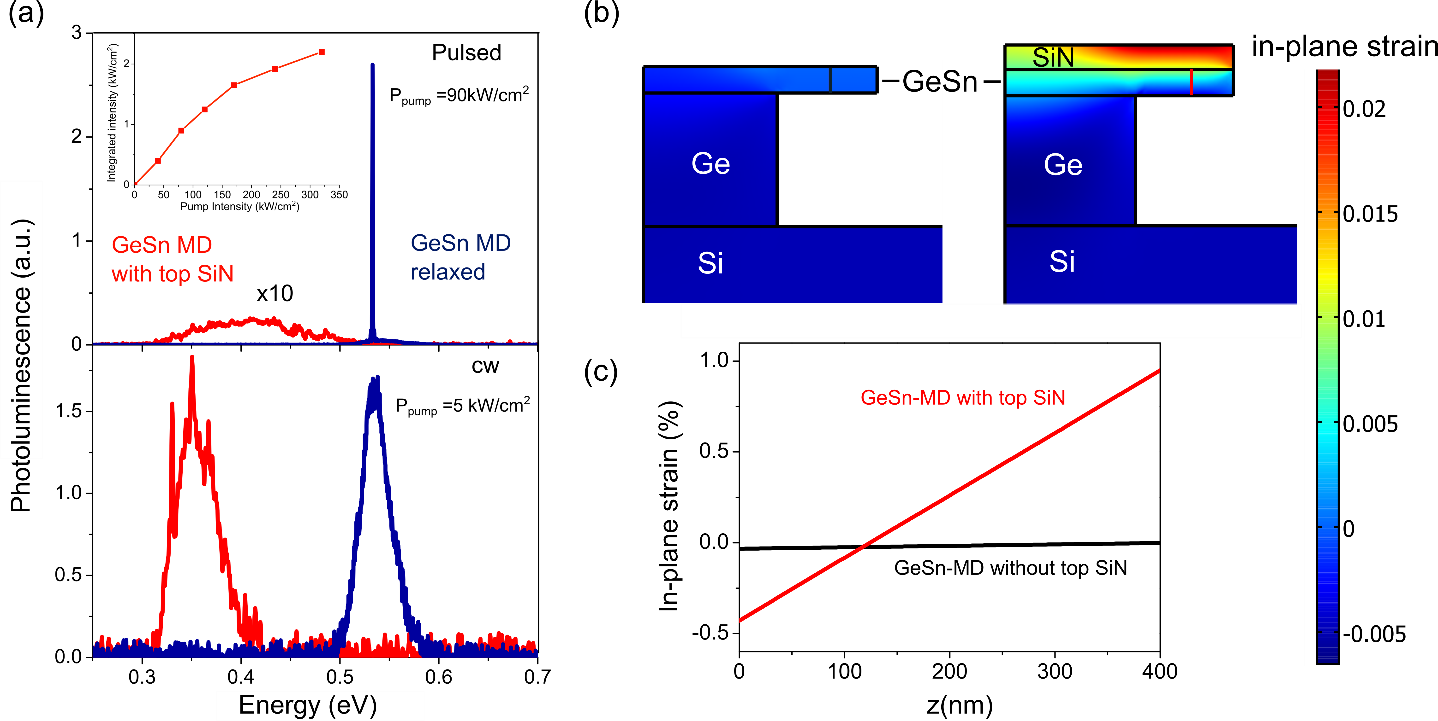
**

Figure S10. (a) PL spectra of 7 µm diameter GeSn microdisks with and without top SiN stressors, under continuous wave (cw) excitation with a density of 5 kW.cm^-2^ (bottom), and under pulsed excitation with a density of 90 kW.cm^-2^ (top). Lasing was reached in the strain-free GeSn microdisk without a top SiN stressor. In contrast, the very same microdisk did not feature optical gain anymore after top SiN desposition. The inset is the L-L curve for the GeSn microdisk with top SiN, without any threshold effect. (b) Cross-section 2D map of in-plane strain distribution in the microdisk volume with and without top SiN stressors on the free-standing GeSn microdisks. (c) in-plane strain distribution along the GeSn layer, from bottom at z = 0 to top at z = 400 nm, at 0.75 µm from the disk periphery for the relaxed GeSn microdisk layer without SiN on top (black) and with a SiN stressor on top (red).

**G-WGM analysis, grating parameters optimization**

This section aims at giving details on the external grating design used to redirect the WGM in-plane radiation pattern into a 40° solid angle. Calculations were performed with an aperiodic Fourier modal method dedicated to body-of-revolution objects [7]. By considering a ring source, the complex frequencies ω= ω**_r_**+iω**_i_** of the modes associated to one given diameter microdisk without Al grating were first calculated. Further simulations showed that the frequency position ω**_r_** of these modes and their quality factor Q=- ω_r_/(2ω_i_**)** were not modified by the addition of an aluminum grating. Thus, a mode calculated for the microdisk without grating, which was matching the experimental spectral frequency, was selected and its diffusion diagram calculated for various circular diffraction gratings surrounding the GeSnOI mesas. Several rings, duty cycles, and periods were simulated in order to maximize the redirection of the WGMs light. The optimal period and duty cycle for the mode TE_13,1_ of a 4-µm-diameter microdisk were respectively 3.4 µm and 90%. It was coherent with a redirection of the light into the -1 diffraction order of the grating. Indeed, as shown on Fig. 7a, the maximal radiation angle of the mode TE_13,1_ (λ_mode_ ≈ 2.3 µm or 0.54 eV) is situated around θ^(mode)^ ≈ 75° with respect to the normal incidence. Thus, according to the grating law (indicated below) applied with d = 4 µm, one obtains θ^(-1)^ ≈ 17°, a coherent value to maximize the radiation pattern into a 40° solid angle.

$$d=\frac{\lambda_{mode}}{\sin\theta^{(mode)}-\sin\theta^{(-1)}}$$

The grating finally taken for the experiments had a lower duty cycle (50%), due to practical fabrication issues. However, the final experimental grating works along the same principle.

**References**

1. A. Elbaz, R. Arefin, E. Sakat, B. Wang, E. Herth, G. Patriarche, A. Foti, R. Ossikovski, S. Sauvage, X. Checoury, K. Pantzas, I. Sagnes, J. Chrétien, L. Casiez, M. Bertrand, V. Calvo, N. Pauc, A. Chelnokov, P. Boucaud, F. Boeuf, V. Reboud, J.-M. Hartmann, and M. El Kurdi, "Reduced Lasing Thresholds in GeSn Microdisk Cavities with Defect Management of the Optically Active Region," ACS Photonics **7**, 2713–2722 (2020).

2. J. Aubin, J.-M. Hartmann, A. Gassenq, J. L. Rouviere, E. Robin, V. Delaye, D. Cooper, N. Mollard, V. Reboud, and V. Calvo, "Growth and structural properties of step-graded, high Sn content GeSn layers on Ge," Semiconductor Science and Technology **32**, 094006 (2017).

3. D. Imbrenda, R. Hickey, R. A. Carrasco, N. S. Fernando, J. VanDerslice, S. Zollner, and J. Kolodzey, "Infrared dielectric response, index of refraction, and absorption of germanium-tin alloys with tin contents up to 27% deposited by molecular beam epitaxy," Applied Physics Letters **113**, 122104 (2018).

4. A. Elbaz, M. El Kurdi, A. Aassime, S. Sauvage, X. Checoury, I. Sagnes, C. Baudot, F. Boeuf, and P. Boucaud, "Germanium microlasers on metallic pedestals," APL Photonics **3**, 106102 (2018).

5. M. El Kurdi, M. Prost, A. Ghrib, S. Sauvage, X. Checoury, G. Beaudoin, I. Sagnes, G. Picardi, R. Ossikovski, and P. Boucaud, "Direct Band Gap Germanium Microdisks Obtained with Silicon Nitride Stressor Layers," ACS Photonics **3**, 443–448 (2016).

6. A. Ghrib, M. El Kurdi, M. de Kersauson, M. Prost, S. Sauvage, X. Checoury, G. Beaudoin, I. Sagnes, and P. Boucaud, "Tensile-strained germanium microdisks," Applied Physics Letters **102**, 221112 (2013).

7. A. Ghrib, M. El Kurdi, M. Prost, S. Sauvage, X. Checoury, G. Beaudoin, M. Chaigneau, R. Ossikovski, I. Sagnes, and P. Boucaud, "All-Around SiN Stressor for High and Homogeneous Tensile Strain in Germanium Microdisk Cavities," Advanced Optical Materials **3**, 353–358 (2015).

8. F. Bigourdan, J.-P. Hugonin, and P. Lalanne, "Aperiodic-Fourier modal method for analysis of body-of-revolution photonic structures," J. Opt. Soc. Am. A **31**, 1303–1311 (2014).
